# Integrated multi-omics profiling of the early post-infarct heart reveals a hub gene network associated with myeloid-driven inflammation

**DOI:** 10.3389/fcvm.2026.1837094

**Published:** 2026-07-13

**Authors:** Zeyang Wang, Jinhu Shi, Yinchuan Lai, Song Wang

**Affiliations:** 1 Department of Cardiology, General Hospital of Central Theater Command of PLA, Wuhan, China; 2 Department of Cardiology, The Second Affiliated Hospital of Chongqing Medical University, Chongqing, China; 3 Hubei Provincial Hospital of Traditional Chinese Medicine, Wuhan, China

**Keywords:** acute myocardial infarction, hub genes, immune infiltration, inflammatory-related genes, RNA-seq, single-cell RNA sequencing

## Abstract

**Background:**

The early inflammatory phase (EIP, 0–72 h) post-acute myocardial infarction (AMI) is a critical determinant of cardiac repair and clinical outcomes. However, a comprehensive understanding of the regulatory gene networks and cellular interactions that govern this decisive period remains incomplete. This study aimed to define the key transcriptional programs and immune dynamics during the EIP of AMI.

**Methods:**

We performed an integrated analysis of time-series bulk RNA-sequencing (RNA-seq) datasets (GSE206281, GSE153494) from murine myocardium post-ischemia. Single-cell RNA-seq (scRNA-seq) data (GSE163129, GSE163465) from cardiac immune cells were analyzed to characterize cellular heterogeneity and intercellular communication. A multi-modal framework combining inflammatory progression scoring with weighted gene co-expression network analysis (WGCNA) was applied to identify critical modules. Hub genes were pinpointed through protein-protein interaction network analysis and intersection with inflammation-related genes (IRGs). Their association with immune infiltration was assessed, and expression was validated in an external dataset, a murine AMI model, and a human peripheral blood cohort (GSE60993).

**Results:**

Temporal analysis identified 160 dynamically regulated genes post-AMI, prominently enriched in myeloid leukocyte activation and extracellular matrix organization. scRNA-seq revealed a remodeled immune landscape at day 3, characterized by increased proportions of macrophages, monocytes, and neutrophils, alongside enhanced intercellular signaling via pathways such as MIF and GALECTIN. Network analysis distilled a core set of seven inflammation-associated hub genes (*Grn*, *Igf1*, *Il18*, *Itgb2*, *Ncf2*, *Ncf4*, *Spp1*). These genes showed cell-type-specific expression patterns in myeloid subsets, correlated positively with myeloid cell infiltration in bulk tissue, and were significantly upregulated in the murine AMI model. Exploratory single-gene ROC analyses in a human peripheral blood cohort suggested preliminary differential expression trends for some hub genes (e.g., Spp1, Ncf4), but the very limited sample size precluded reliable construction of a multi-gene model and renders these findings strictly hypothesis-generating.

**Conclusions:**

This study delineates a high-resolution map of transcriptional and cellular dynamics during the EIP of AMI, revealing a coordinated network of inflammatory mediators linked to early myeloid cell recruitment and activation. The identified seven-candidate hub genes represents a prioritized set of candidates for future investigation into diagnostic biomarkers and therapeutic strategies targeting the early inflammatory window.

## 1 Background

Cardiovascular disease mortality has risen steadily worldwide, driven by population aging, with acute myocardial infarction (AMI) accounting for the vast majority of cardiac deaths (1). The pathophysiology of ischemic injury encompasses cardiomyocyte apoptosis, activation of inflammatory signaling, reactive oxygen species generation, and dysregulation of immune pathways, ultimately leading to excessive myocardial damage and subsequent heart failure (2). Post-AMI cardiac repair is a staged process, and the initial inflammatory phase is widely recognized as a critical determinant of subsequent remodeling and clinical outcomes (3, 4). Specifically, the early inflammatory phase (EIP, within the first 72 h post-AMI) represents a decisive window where a coordinated yet contained inflammatory response is essential for clearing debris and initiating repair (5–7). Conversely, excessive, prolonged, or dysregulated inflammation during this phase can drive adverse ventricular remodeling, irreversible fibrotic scarring, and progression to heart failure (8, 9). Therefore, gaining a precise understanding of the regulatory networks and cellular dynamics within this narrow therapeutic window is paramount for developing strategies to modulate post-AMI inflammation.

Continuous efforts have been made to characterize the inflammatory processes during the EIP (4, 6, 9). For instance, inhibition of Lgr4 (leucine-rich repeat-containing G protein-coupled receptor 4) during the EIP confers cardioprotection by reducing leukocyte infiltration, linked to the regulation of AP-1 activity (10). Dusp6 and Dectin-1 have been reported to modulate neutrophil-mediated damage in the early phase (11, 12). Transcriptomic studies have profiled changes in myocardial tissue post-AMI (13, 14), and single-cell RNA sequencing (scRNA-seq) has begun to unravel the functional heterogeneity of cardiac cells after injury (15–17). However, a systematic integration of temporal bulk transcriptomic dynamics with single-cell resolution characterization of the immune microenvironment during the critical 0–72 h window is lacking. Furthermore, the key molecular hubs that coordinate the early immune response and their cell-type-specific expression patterns remain insufficiently elucidated.

To address these gaps, we integrated multiple bulk and single-cell RNA-seq datasets capturing the early phase of AMI. We first identified time-dependent gene expression trends post-ischemia. We then combined inflammatory progression scoring with weighted gene co-expression network analysis (WGCNA) on scRNA-seq data to pinpoint gene modules and hub genes associated with the early immune response. The identified inflammatory hub genes were validated in an external dataset, a murine AMI model, and their association with clinical outcomes was assessed. Our study provides a multi-dimensional transcriptomic map of the EIP, identifying pivotal inflammatory mediators and offering insights into potential diagnostic markers and therapeutic targets for modulating the early inflammatory response in AMI.

## 2 Materials and methods

### 2.1 Integration and temporal trend analysis of bulk RNA-seq data

Two publicly available mouse myocardial time-series RNA-seq datasets (GSE206281 and GSE153494), both profiling the left ventricle at six time points post-AMI (Sham/Control, 10 min, 1 h, 6 h, 24 h, and 72 h) with three biological replicates per time point (13, 14), were downloaded from the GEO database. Raw count matrices were processed and normalized independently using the appropriate pipeline for each dataset (as described in their original publications). To enable joint analysis, normalized expression matrices from both datasets were merged, and the ComBat function from the sva R package (v3.44.0) was applied to adjust for technical batch effects while preserving biological variation related to time points. The resulting integrated expression profile contained 36 samples. Principal component analysis (PCA) before and after batch correction confirmed that samples clustered primarily by time point rather than dataset origin (Figure S1A), indicating robust biological signals across studies. To comprehensively capture the temporal dynamics of gene expression during the early phase post-AMI, we performed time-series clustering on the integrated dataset using all expressed genes. Prior to clustering, genes with low expression or missing values were filtered using the filter.NA function (thres = 0.25) in the Mfuzz package (v2.56.0). Subsequently, genes with low variance across the time course (min.std = 0.5) were removed using the filter.std function to focus on dynamically expressed genes. Fuzzy c-means clustering was then performed on the remaining genes to identify clusters of genes sharing similar expression trajectories across the six time points. Gene Ontology (GO) and Kyoto Encyclopedia of Genes and Genomes (KEGG) pathway enrichment analyses for the identified clusters were performed using the clusterProfiler package (v4.4.4). *P*-values were adjusted using the Benjamini-Hochberg (BH) method.

### 2.2 Processing of single-cell RNA sequencing (scRNA-Seq) data

Single-cell RNA-seq data were obtained from two studies focusing on cardiac immune cells post-MI (15, 18). The Sham samples (GSM4972357, GSM4985022) and MI-3day samples (GSM4972359, GSM4985023) were sourced from datasets GSE163129 and GSE163465, respectively. Data processing was performed using the Seurat package (v4.1.0). Cells were filtered based on the following quality control thresholds: genes detected per cell >200, total transcripts per cell >1,000, and mitochondrial gene ratio <15%. Potential doublets were identified and removed using DoubletFinder (v2.0.3) with parameters pN = 0.25 and a pK value determined empirically for each sample. After filtering, the datasets were integrated using the FindIntegrationAnchors and IntegrateData functions to correct for batch effects. The integrated dataset contained 22,147 high-quality cells.

Normalization, scaling, and dimensionality reduction were performed on the integrated data. Principal component analysis (PCA) was conducted on the top 2,000 highly variable genes. The top 20 principal components were used for downstream clustering and uniform manifold approximation and projection (UMAP) visualization. Cell clusters were identified using the FindNeighbors and FindClusters functions (resolution = 0.5). Major immune cell types were annotated manually based on the expression of canonical marker genes: macrophages (*C1qa*, *C1qb*), monocytes (*Ly6c2*, *Plac8*), neutrophils (*S100a8*, *S100a9*), dendritic cells (*Cd209a, H2−DMb1*), B cells (*Ebf1*, *Cd79a*), T cells (*Cd3d*, *Cd3e*), and natural killer (NK) cells (*Nkg7*, *Trbc2*).

### 2.3 Analysis of cell–cell communication

To infer intercellular communication networks within the cardiac immune microenvironment, we applied the CellChat R package (v1.6.1) to the integrated and annotated scRNA-seq data. The analysis was run separately on the Sham and MI-3day groups. CellChat utilizes a curated database of ligand-receptor interactions to calculate communication probabilities and identify significant signaling pathways. The net difference in interaction strength and information flow between the two conditions was computed to reveal pathways altered post-MI. Significant pathways were identified based on a permutation test, with *P*-values adjusted using the BH method.

### 2.4 Construction of weighted gene co-expression networks and inflammatory scoring

An inflammatory progression score (IPS) was calculated for each single cell using the AddModuleScore function in Seurat, based on the combined gene lists from Mfuzz Clusters 4, 5, and 6, which showed sustained upregulation. Although Cluster 4 genes are enriched for extracellular matrix (ECM) organization, ECM remodeling is intrinsically linked to the inflammatory response, as leukocyte extravasation and migration require ECM degradation and restructuring. Thus, inclusion of Cluster 4 genes captures the structural aspects of the inflammatory microenvironment. To perform Weighted Gene Co-expression Network Analysis (WGCNA) on single-cell data while mitigating sparsity, we used the hdWGCNA package (v0.2.12). Metacells were generated from the myeloid subsets (macrophages and monocytes with high IPS) using the MetacellsByGroups function (*k* = 20, max_shared = 10), grouping by sample condition. The parameter *k* = 20 was selected based on the default recommendation of the hdWGCNA package for datasets of similar cellularity, balancing noise reduction against preservation of biological heterogeneity. The metacell expression matrix was normalized, scaled, and Harmony was applied to correct for residual technical effects. A soft power threshold of 5 was selected using the TestSoftPower function to achieve a scale-free topology fit >0.8. A signed hybrid network was constructed using the ConstructNetwork function. Module eigengenes were calculated, and the correlation (kME) of each gene to its module eigengene was determined.

### 2.5 Identification of hub genes associated with inflammation

The top 150 genes ranked by kME (module connectivity) from the two MI-associated modules (Modules 5 & 6) were selected. The overlap between these 160 unique genes and the genes from Mfuzz Clusters 4–6 was identified. To extract functional clusters, a protein-protein interaction (PPI) network for these 160 genes was constructed using the STRING database (minimum interaction score: 0.400) and visualized in Cytoscape (v3.9.1). The Molecular Complex Detection (MCODE) plugin was applied to this network using default parameters (Degree Cutoff: 2, Node Score Cutoff: 0.2, K-Core: 2, Max. Depth: 100) to identify the most significant cluster of densely connected genes. Functional enrichment analysis of this MCODE cluster was performed. To assess the robustness of the core module, we additionally constructed a PPI network using a higher confidence threshold (score ≥0.700) and confirmed that the MCODE-identified core cluster remained highly consistent. A separate list of inflammation-related genes (IRGs) was obtained from the GeneCards database (https://www.genecards.org), filtered for a high-confidence relevance score > 5. This threshold was selected to balance sensitivity and specificity, yielding a broad yet functionally coherent set of inflammation-associated genes. To further validate this selection, we cross-referenced the seven genes against the MSigDB hallmark gene set “Inflammatory Response” and confirmed their inclusion in canonical inflammatory pathways. The final seven hub inflammatory genes were defined as the intersection between the genes in the core MCODE cluster and this high-confidence IRG list.

### 2.6 Transcription factor network and functional enrichment of hub genes

Potential transcriptional regulators of the seven hub genes were predicted using the JASPAR2022 database via the RcisTarget and AUCell packages. Transcription factor (TF)-target gene interactions with a normalized enrichment score (NES) > 3.0 and a recovery rate of the TF binding motif in the gene's regulatory region >8% were considered significant and used to construct a regulatory network. Gene Set Enrichment Analysis (GSEA) and Gene Set Variation Analysis (GSVA) were performed on pre-ranked gene lists (based on correlation with each hub gene's expression) using hallmark and KEGG gene sets from the MSigDB collections. *P*-values were adjusted using the BH method.

### 2.7 Immune cell infiltration and correlation analysis

The relative abundance of 29 immune cell types in the bulk RNA-seq samples (integrated GSE206281/GSE153494) was estimated using the web tool ImmuCellAI (http://bioinfo.life.hust.edu.cn/ImmuCellAI/), which employs a gene set signature-based method. We acknowledge that ImmuCellAI was originally developed using human immune cell signatures. To apply it to murine data, we mapped human immune marker genes to their mouse orthologs using the Ensembl BioMart database (GRCm38) prior to analysis. The infiltration scores between Sham and MI-3day groups were compared using the Wilcoxon rank-sum test, with *P*-values adjusted using the BH method. The correlation between the expression of each of the seven hub genes and the infiltration scores of various immune cells was calculated using Spearman's rank correlation coefficient, with *P*-values adjusted for multiple testing using the BH method.

### 2.8 External datasets for validation

For independent mRNA validation, the mouse myocardial infarction dataset GSE161427 was used (19). Normalized expression data for the Sham (*n* = 4) and 3-day post-MI (*n* = 5) groups were extracted, and the expression levels of the seven hub genes were compared.

For exploratory clinical assessment, the human peripheral blood mononuclear cell (PBMC) RNA-seq dataset GSE60993 was utilized (15). This dataset includes patients with ST-elevation myocardial infarction (STEMI, *n* = 7), non-STEMI (NSTEMI, *n* = 10), unstable angina (UA, *n* = 9), and healthy controls (*n* = 7). Due to the very small sample size, we refrained from constructing multi-gene logistic regression models to avoid overfitting. Instead, we evaluated the individual diagnostic performance of each hub gene using receiver operating characteristic (ROC) curve analysis with the pROC package. To provide a robust estimate of uncertainty, we calculated 95% confidence intervals (CIs) for each area under the curve (AUC) using 1,000 bootstrap iterations. These analyses were performed separately for the entire cohort (all AMI patients vs. controls) and for the STEMI subgroup (STEMI vs. controls). All results are presented as exploratory findings requiring independent validation.

### 2.9 Experimental animals and myocardial infarction model

All animal procedures were approved by the Animal Ethics Committee of The Second Affiliated Hospital of Chongqing Medical University (Approval No. IACUC-SAHCQMU-2024-00115) and complied with institutional and national guidelines. Male C57BL/6J mice (10–12 weeks old, 22–26 g) were randomly assigned to sham-operated control or myocardial infarction (MI) groups using a computer-generated randomization sequence. For the molecular analyses presented in this study (qRT-PCR and Western blot), a cohort of 6 mice per group was used. The MI model was established by permanent ligation of the left anterior descending (LAD) coronary artery. Sham mice underwent the same procedure without ligation. All surgical procedures were performed by a single investigator who was not blinded to group allocation due to the nature of the surgery; however, echocardiographic measurements and molecular analyses were conducted by independent investigators blinded to group assignment. The success of the AMI model was confirmed by significant impairment of cardiac function assessed via echocardiography at day 3 post-surgery. For molecular analyses, mice were sacrificed 3 days post-operation. Myocardial tissue was collected from the peri-infarct border zone, as this region represents the active front of the early inflammatory and reparative response central to our study. Tissues were snap-frozen for subsequent RNA and protein extraction.

### 2.10. Echocardiography

Transthoracic echocardiography was performed on lightly anesthetized mice (1.5% isoflurane) using a Vevo 3100 imaging system (VisualSonics, Toronto, Ontario, Canada) with a 30 MHz transducer. Left ventricular (LV) parameters, including end-diastolic and end-systolic internal diameters (LVIDd, LVIDs), were measured from M-mode tracings in the parasternal short-axis view at the papillary muscle level. Left ventricular ejection fraction (LVEF) and fractional shortening (LVFS) were calculated using the system's software.

### 2.11. RNA extraction, quantitative real-time PCR (qRT-PCR), and western blotting

Total RNA was extracted from homogenized peri-infarct tissue using TRIzol reagent (Invitrogen). cDNA was synthesized using the PrimeScript RT Reagent Kit (Takara). qRT-PCR was performed with SYBR Green Master Mix (Roche) on a QuantStudio 5 system (Applied Biosystems). Gene expression was normalized to *Gapdh* and calculated using the 2^−ΔΔCt^ method. Primer sequences are listed in Supplementary Table S1.

For western blotting, tissue proteins were extracted with RIPA buffer containing protease and phosphatase inhibitors. Equal amounts of protein (30 µg) were separated by SDS-PAGE and transferred to PVDF membranes. Membranes were blocked and incubated overnight at 4 °C with primary antibodies against GRN (ab187070, Abcam), IGF1 (ab9572), IL18 (ab207323), ITGB2 (ab52920), NCF2 (ab175293), NCF4 (ab231639), SPP1 (ab166709), and GAPDH (ab8245). After incubation with HRP-conjugated secondary antibodies, signals were detected using an ECL substrate and visualized with a ChemiDoc XRS+ system (Bio-Rad). Densitometric analysis was performed using ImageJ software (NIH), and protein levels were normalized to GAPDH.

### 2.12. Statistical analysis

For bioinformatics analyses, statistical tests are described in their respective sections above. For experimental data, results are presented as mean ± standard error of the mean (SEM). Differences between two groups were assessed using an unpaired two-tailed Student's *t*-test or the Mann–Whitney *U* test if data were not normally distributed. A *p*-value <0.05 was considered statistically significant. All statistical analyses for experimental validation were performed using GraphPad Prism software (v9.0). The R scripts used for data processing, analysis have been provided as Supplementary File 1 (Code_Scripts.rar).

## 3 Results

### 3.1 Temporal transcriptomic dynamics identify inflammation and repair pathways activated during the early phase of AMI

To systematically characterize the molecular transitions during the critical early phase post-AMI, we integrated two time-series bulk RNA-seq datasets (GSE206281, GSE153494), encompassing six time points from 10 min to 72 h after ischemia. Batch effect correction successfully harmonized the datasets, as visualized by PCA (Figure S1A). Hierarchical clustering confirmed that samples grouped primarily by time point rather than dataset origin, indicating robust biological signals across studies (Figure S1B).

**Figure 1:**
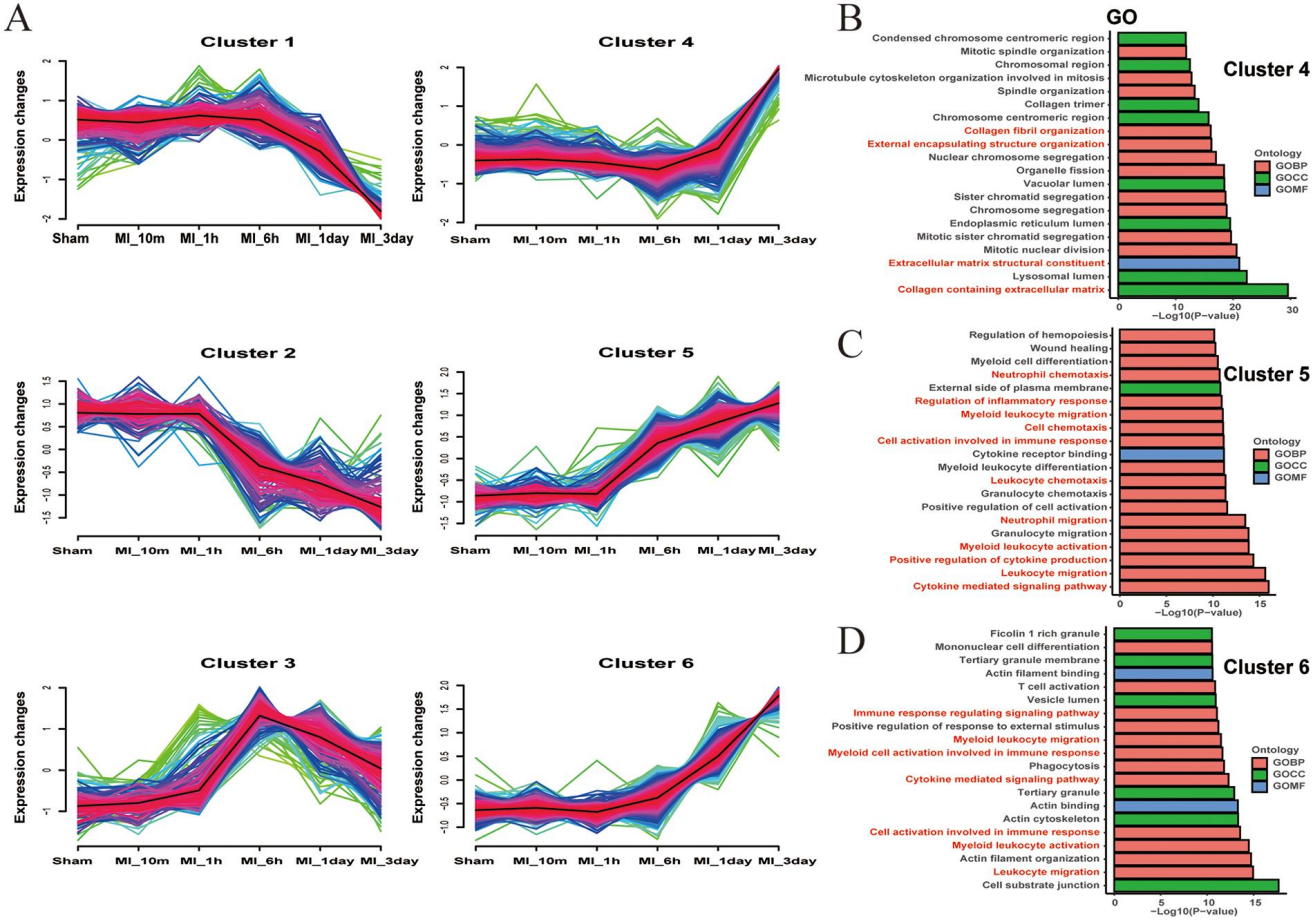
Temporal transcriptomic dynamics during the early phase of AMI. **(A)** Six distinct gene expression trajectory clusters identified by fuzzy c-means clustering (Mfuzz) across six time points post-AMI. The clusters contain 238 (Cluster 1), 160 (Cluster 2), 189 (Cluster 3), 785 (Cluster 4), 355 (Cluster 5), and 862 (Cluster 6) genes, respectively. **(B–D)** Gene Ontology (GO) enrichment analysis of the biologically relevant clusters 4, 5, and 6, respectively.

To map the global transcriptional dynamics, we performed fuzzy c-means clustering (Mfuzz) on filtered genes across the time series. This analysis revealed six distinct clusters of genes characterized by shared expression trajectories (Figure 1A). Clusters 4, 5, and 6 exhibited a sustained upward trend over the 72-h window. Notably, genes in Cluster 5 showed an earlier initiation of upregulation (by 1 h), while genes in Clusters 4 and 6 increased notably by 6 h post-AMI, suggesting a coordinated yet phased activation of biological programs.

Functional enrichment analysis provided mechanistic context for these dynamic clusters. Cluster 4 genes were predominantly associated with extracellular matrix organization, collagen fibril assembly, and structural constituent of the ECM (Figure 1B), indicating early activation of tissue repair and remodeling processes. In contrast, Clusters 5 and 6 were overwhelmingly enriched for immune and inflammatory functions. Cluster 5 terms included myeloid leukocyte activation, cell activation involved in immune response, and regulation of inflammatory response (Figure 1C). Cluster 6 shared similar themes, highlighting myeloid leukocyte migration, chemotaxis, and cytokine-mediated signaling pathways (Figure 1D). KEGG pathway analysis reinforced these findings, identifying Chemokine signaling pathway, Cytokine-cytokine receptor interaction, and ECM-receptor interaction as key pathways enriched across these clusters (Supplementary Figures S1C–E). This sequential and overlapping activation of inflammatory and reparative pathways underscores the integrated and dynamic nature of the early response to myocardial ischemia.

### 3.2 Single-sell profiling unveils immune microenvironment remodeling and enhanced intercellular communication post-AMI

**Figure 2:**
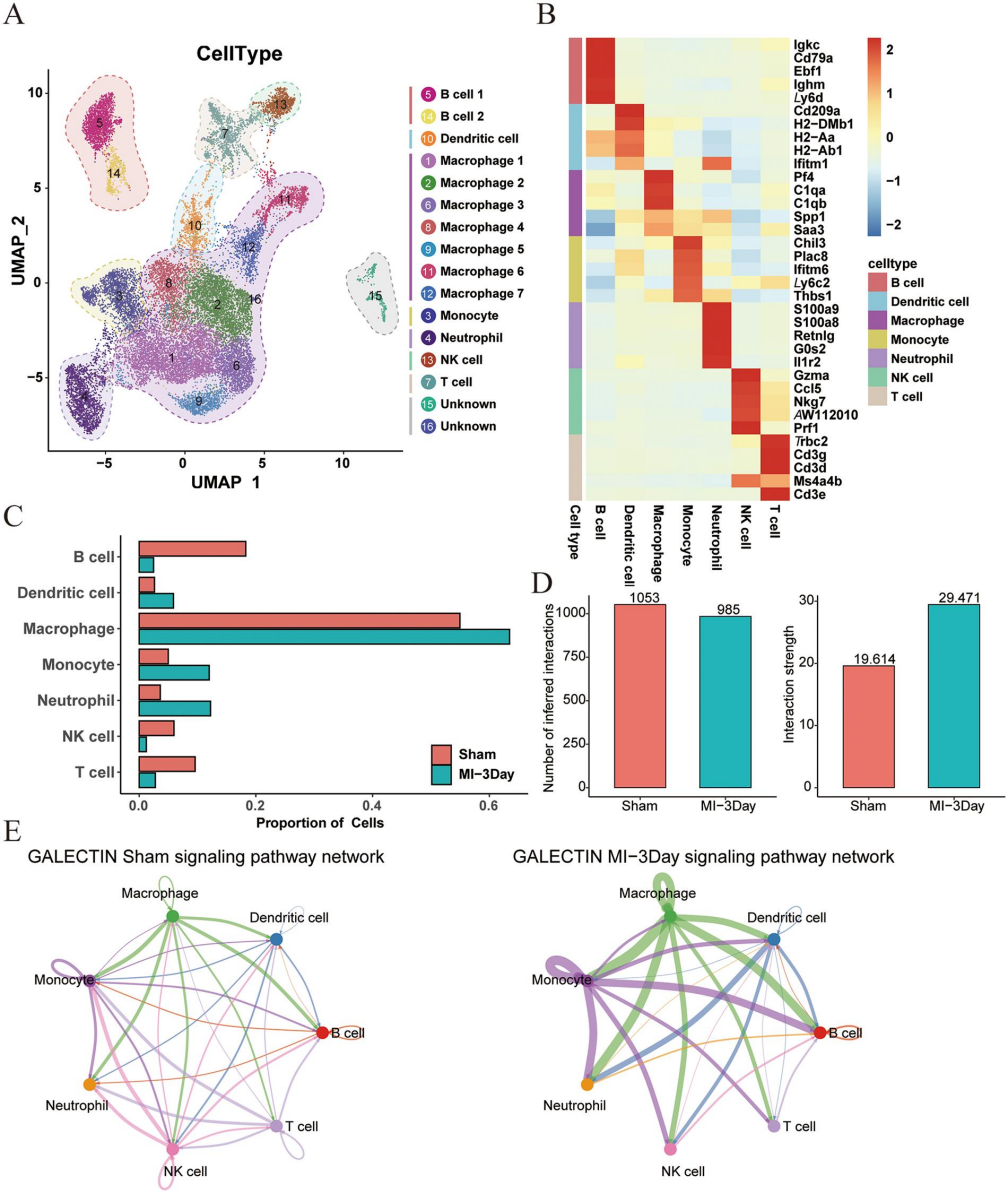
Single-cell profiling of the cardiac immune microenvironment post-AMI. **(A)** UMAP visualization showing seven major immune cell types annotated based on canonical marker genes. **(B)** Heatmap displaying the expression of the top five marker genes for each immune cell type. **(C)** Bar plot comparing the relative proportion of each immune cell type between Sham and MI-3day groups.**(D)** The total number and strength of cell–cell interactions inferred by CellChat in each group. **(E)** Circle plot illustrating the differential activity of the GALECTIN signaling pathway between cell types in the MI-3day vs. Sham groups.

To deconvolve the cellular underpinnings of the bulk transcriptomic shifts, we analyzed integrated scRNA-seq data from CD45+ immune cells isolated from Sham and 3-day post-MI hearts. We acknowledge that the scRNA-seq data only provide a snapshot at day 3 post-MI; thus, the temporal dynamics described here rely primarily on the bulk time-series data. After rigorous quality control and batch integration via Harmony, UMAP visualization revealed clear separation between conditions and identified 16 transcriptionally distinct clusters (Figure 2A; Supplementary Figures S2A,B). Annotation using canonical markers defined seven major immune lineages: macrophages, monocytes, neutrophils, dendritic cells, B cells, T cells, and natural killer (NK) cells (Figures 2A,B). A heatmap displaying the expression levels of the top five marker genes for each of these seven immune cell types is presented in Figure 2B.

Interrogation of cellular composition revealed a profound remodeling of the cardiac immune landscape at day 3 post-MI (Figure 2C). This was characterized by a marked expansion of cells from the myeloid lineage: the proportion of neutrophils increased from 3.56% (Sham) to 11.94%, monocytes from 4.91% to 11.72%, and macrophages from 54.14% to 61.99%. Concomitantly, the relative proportions of lymphocyte subsets decreased (B cells: 18.0% to 2.41%; T cells: 9.44% to 2.7%; NK cells: 5.87% to 1.19%), likely reflecting the massive influx of myeloid cells altering the overall cellular census.

To move beyond static composition and understand dynamic interactions, we performed cell–cell communication analysis using CellChat. This revealed a significant global enhancement in strength of inferred ligand-receptor interactions in the MI-3day group compared to Sham, but a little decease in the number of interaction (Figure 2D). Pathway-level analysis identified several inflammatory signaling networks that were differentially active (Figure S2C). Notably, signaling pathways such as THBS, GALECTIN, and FN1 exhibited markedly increased activity post-MI (Figure 2E; Supplementary Figure S2D). THBS, GALECTIN, and FN1 are known to mediate chemotaxis, adhesion, and pro-inflammatory activation among monocytes, macrophages, and neutrophils (20, 21). This analysis suggests that the early post-infarct period is not only defined by changing cellular abundances but also by intensified and altered communicative networks between these immune subsets, potentially driving the inflammatory cascade.

### 3.3 Weighted gene co-expression network analysis pinpoints modules coregulated with inflammatory progression in key myeloid subsets

**Figure 3:**
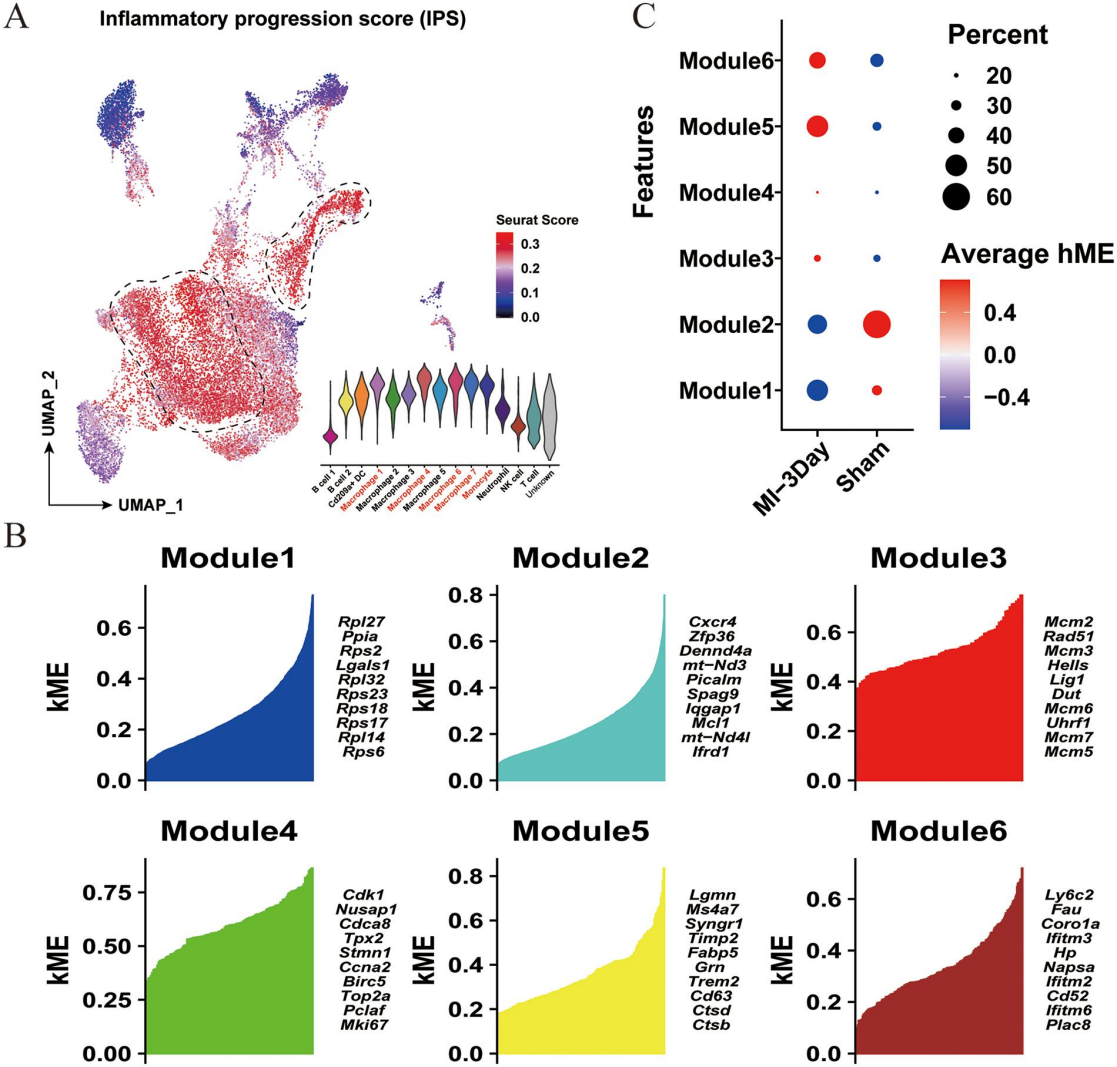
Identification of inflammatory progression-associated gene modules via scRNA-Seq-based WGCNA. **(A)** Left: UMAP projection of the inflammatory progression score (IPS) across all cells. Right: Highlight of the five myeloid subpopulations (macrophages 1,4,6,7 and monocytes) with the highest IPS. **(B)** Heatmap of the top genes ranked by module eigengene (kME) values within each of the six identified co-expression modules.**(C)** Dot plots showing the distribution of harmonized module eigengenes (hMEs) for each module in Sham and MI-3day groups. Modules 5 and 6 show significantly higher hMEs in the MI-3day group.

Given the centrality of myeloid cells, we sought to identify gene programs specifically correlated with the inflammatory state within these populations. We calculated an inflammatory progression score (IPS) for each cell based on genes from Mfuzz Clusters 4–6. As expected, macrophage and monocyte subsets displayed the highest IPS (Figure 3A). To reduce noise for network construction, we aggregated cells into metacells (Figure S3A-B). WGCNA performed on the metacell expression matrix from high-IPS myeloid subsets identified six distinct co-expression modules (Figure 3B; Supplementary Figure S3C). The harmonized module eigengenes (hMEs) for modules 5 and 6 were significantly elevated in metacells derived from the MI-3day group compared to Sham (Figure 3C), marking them as transcriptional modules strongly associated with the post-infarction inflammatory state.

### 3.4 A multi-step bioinformatics pipeline identifies a core network of seven inflammatory hub genes

**Figure 4:**
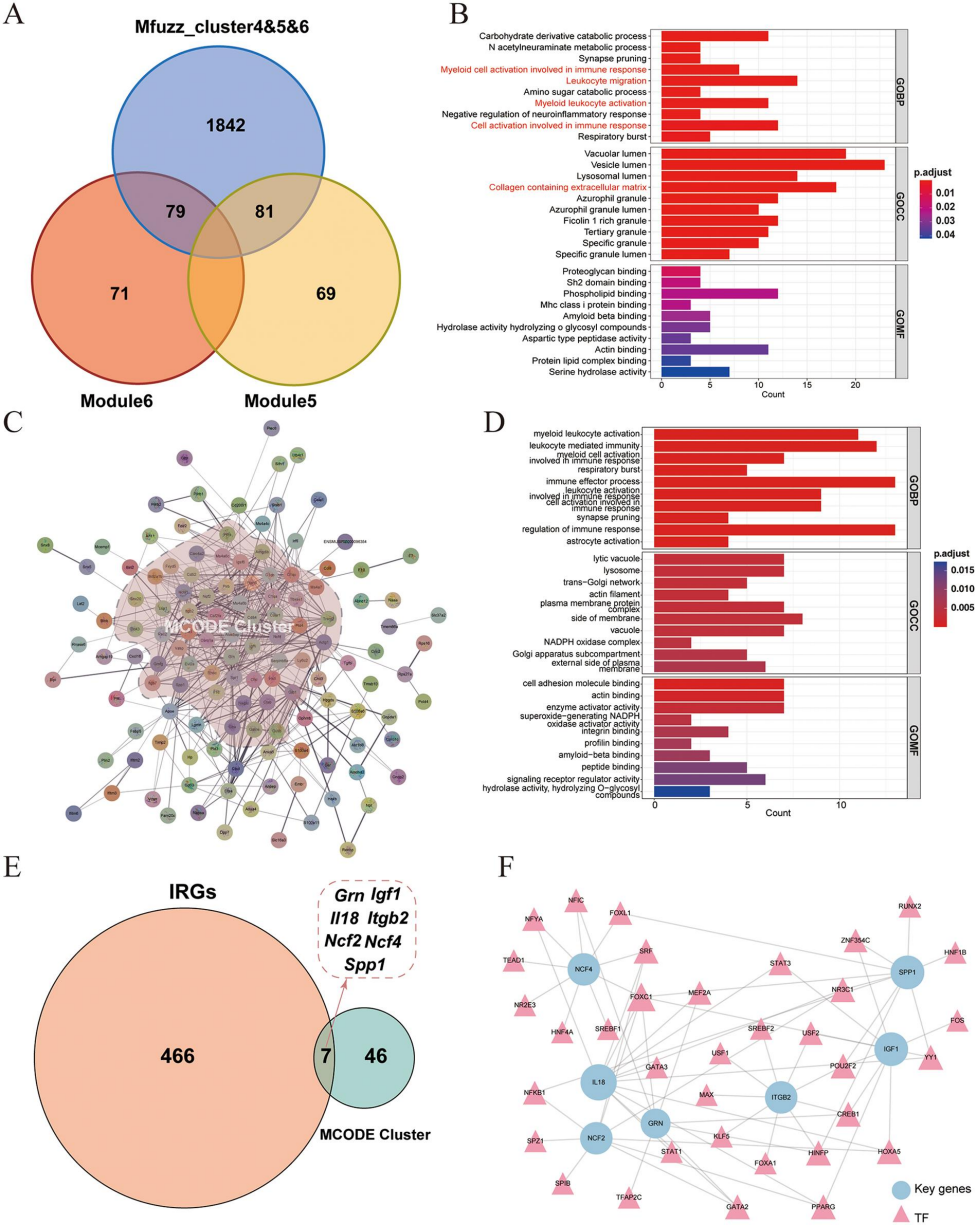
Screening and characterization of hub inflammation-related genes. **(A)** Venn diagrams illustrating the overlap between genes from temporal Mfuzz clusters (4,5,6) and scRNA-Seq WGCNA modules (5 & 6), yielding 160 candidate genes. **(B)** Gene Ontology (GO) enrichment analysis of the 160 candidate genes. **(C)** The protein-protein interaction (PPI) network of the 160 genes, with the core module identified by MCODE highlighted. **(D)** GO enrichment analysis of the 53 genes within the core MCODE cluster. **(E)** Venn diagram identifying the seven final hub genes at the intersection of the MCODE cluster and a high-confidence inflammation-related gene (IRG) set. **(F)** Regulatory network of the seven hub genes and their predicted upstream transcription factors (TFs).

To distill the most critical regulators, we focused on the 160 genes that were both highly connected within MI-associated modules (5 & 6) and part of the temporally upregulated clusters (4–6) (Figure 4A). GO enrichment of these 160 genes reaffirmed their collective role in immune effector processes, leukocyte activation, and response to cytokine (Figure 4B). Constructing a protein-protein interaction (PPI) network and applying the MCODE algorithm extracted a densely interconnected cluster of 53 genes (Figure 4C), which was independently confirmed to be highly enriched for inflammatory GO terms (Figure 4D). To further prioritize genes with direct inflammatory relevance, we intersected this cluster with a high-confidence list of inflammation-related genes (IRGs, relevance score >5 from GeneCards). This stringent filtering yielded seven hub genes: Granulin (Grn), Insulin-like growth factor 1 (Igf1), Interleukin-18 (Il18), Integrin subunit beta 2 (Itgb2), Neutrophil cytosolic factor 2 (Ncf2), Neutrophil cytosolic factor 4 (Ncf4), and Secreted phosphoprotein 1 (Spp1/Osteopontin) (Figure 4E). Network analysis predicted potential upstream transcriptional regulators (Figure 4F), including NF-*κ*B1, FOXC1, and GATA2, known to be involved in immune and stress responses (22–24).

### 3.5 Hub genes exhibit cell-type-specific expression, are coordinately upregulated, and hold diagnostic potential

**Figure 5:**
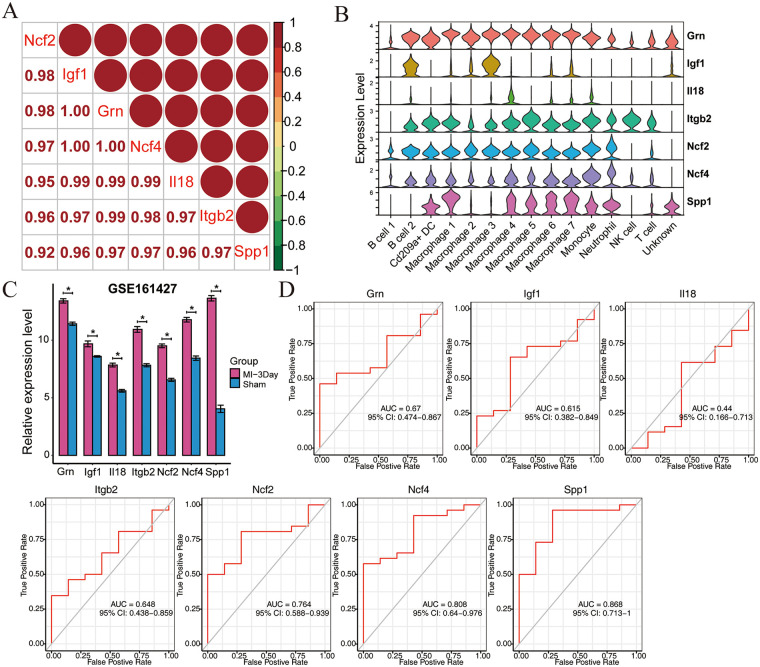
Expression patterns and diagnostic potential of the inflammatory hub genes. **(A)** Correlation matrix showing pairwise Spearman correlation coefficients among the seven hub genes in the integrated bulk RNA-Seq data. **(B)** Violin plots depicting the cell-type-specific expression distribution of each hub gene across immune cell subsets in the scRNA-Seq data. **(C)** Validation of hub gene mRNA expression upregulation in an independent mouse myocardial dataset (GSE161427). Data are presented as mean ± SEM; *p < 0.05, **p < 0.01 (unpaired two-tailed *t*-test). **(D)** Individual receiver operating characteristic (ROC) curves for each hub gene in distinguishing AMI patients (all AMI, *n* = 26) from healthy controls (*n* = 7) in the GSE60993 cohort. Bootstrap 95% confidence intervals (1,000 iterations) are shown in parentheses. Due to the small sample size, these results are exploratory and require independent validation.

The seven hub genes showed significant positive correlations with each other in the bulk data (Figure 5A), suggesting co-regulation or participation in related pathways. Examining their expression at single-cell resolution revealed nuanced, cell-type-specific patterns (Figure 5B; Supplementary Figure S4A). *Itgb2* was broadly expressed across most immune cells, consistent with its role as a common integrin. *Ncf2* and *Ncf4* showed highly correlated expression, primarily in neutrophils and monocytes. *Il18* and *Igf1* expression was largely confined to macrophage and monocyte subsets. *Spp1* was prominently expressed in a subset of macrophages and monocytes. *Grn* was also highly expressed in macrophages (Figure 5B). This detailed map links specific hub genes to particular cellular actors in the early immune response.

To further identify the signaling pathways associated with the progression of AMI, Gene Set Enrichment Analysis (GSEA) and Gene Set Variation Analysis (GSVA) were conducted. The results, detailed in Supplementary Figures 5 and 6, showed that gene sets related to the hub inflammatory molecules were significantly enriched in pathways such as cytokine-cytokine receptor interaction, chemokine signaling pathway, and oxidative phosphorylation.

Validation in an independent mouse myocardial transcriptomic dataset (GSE161427) confirmed significant upregulation of all seven hub genes at 3 days post-MI compared to Sham (Figure 5C).

To explore the translational relevance of these hub genes, we assessed their individual diagnostic performance in a human peripheral blood dataset (GSE60993). Given the small sample size (STEMI *n* = 7, healthy control *n* = 7 for the subgroup analysis), we focused on single-gene ROC analyses with bootstrap 95% confidence intervals (CIs) to mitigate overfitting concerns. For the entire cohort (all AMI patients vs. controls), *Spp1* (AUC = 0.868, 95% CI: 0.713–1.000), *Ncf4* (AUC = 0.808, 95% CI: 0.640–0.976), and *Ncf2* (AUC = 0.764, 95% CI: 0.588–0.939) showed promising individual discriminatory ability, while *Il18* (AUC = 0.440, 95% CI: 0.166–0.713) did not (Figure 5D). In the STEMI subgroup (STEMI vs. controls, *n* = 7 per group), similar trends were observed (Supplementary Figure S7). These results are exploratory and require validation in larger, independent cohorts before any clinical application can be considered.

It is noteworthy that the peripheral blood samples in this validation cohort were obtained at hospital presentation, typically within hours of symptom onset. While this aligns with our focus on the early inflammatory window (0–72 h post-AMI), we acknowledge the inherent variability in precise sampling times. Future prospective studies employing standardized, serial blood sampling from precisely timed intervals are warranted to further validate the temporal dynamics and clinical utility of this inflammatory gene signature.

### 3.6 Hub gene expression correlates with myeloid cell infiltration in the infarcted myocardium

**Figure 6:**
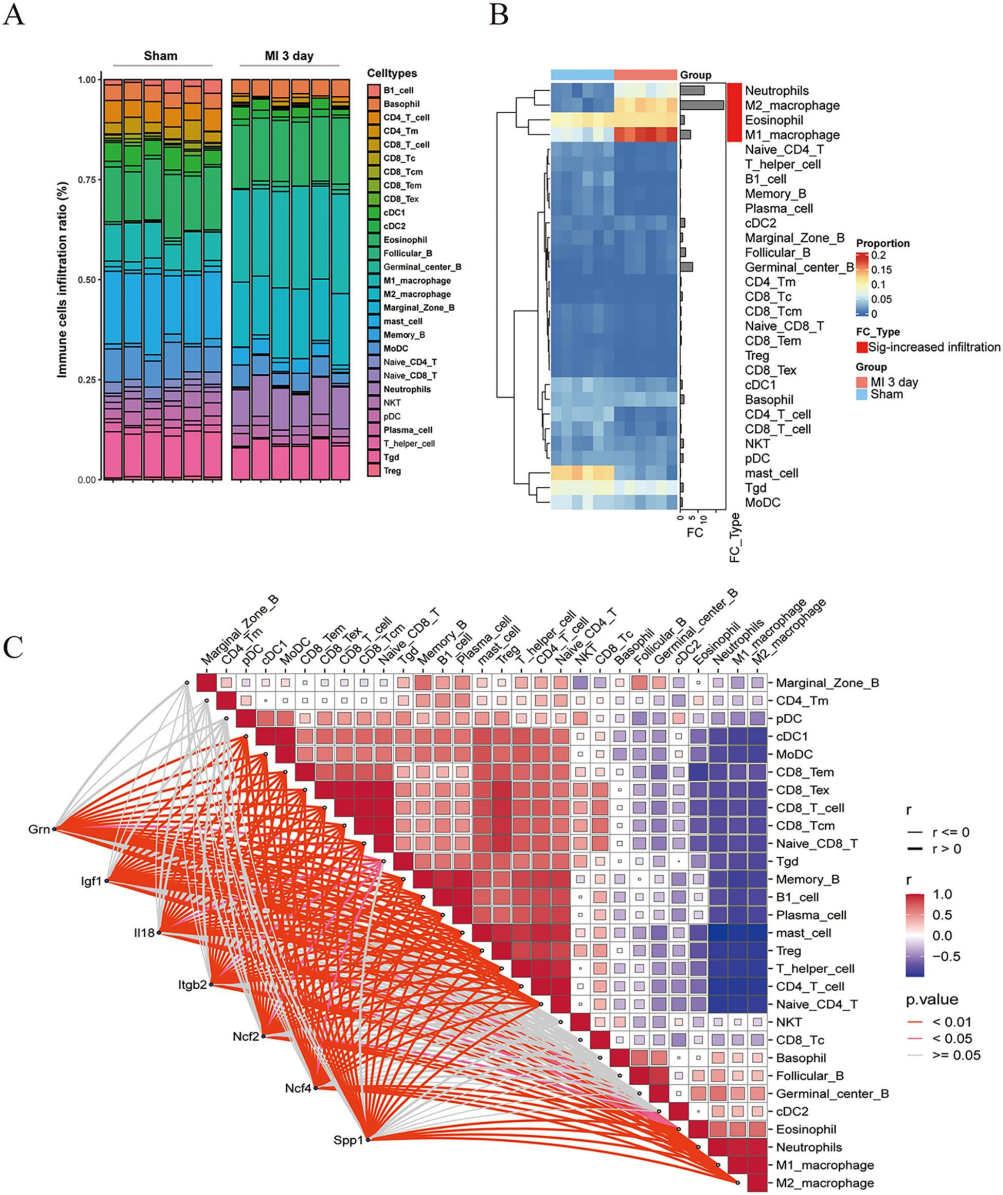
Immune cell infiltration landscape and its correlation with hub genes. **(A)** Stacked bar chart showing the relative abundance of 24 immune cell types estimated by ImmuCellAI in Sham and MI-3day samples from the integrated bulk datasets. **(B)** Heatmap comparing the infiltration scores of significantly altered immune cell types between Sham and MI-3day groups. **(C)** Heatmap of Spearman correlation coefficients between the expression of the seven hub genes and the infiltration scores of various immune cells.

Using the ImmuCellAI deconvolution algorithm on bulk RNA-seq data (GSE206281 and GSE153494), we quantified the infiltration of 29 immune cell types. Consistent with scRNA-seq findings, the MI-3day group showed significantly higher infiltration scores for neutrophils, M1 and M2 macrophages, and eosinophils (Figures 6A,B). Correlation analysis revealed that the expression levels of all seven hub genes were positively associated with the infiltration abundance of these myeloid cells, particularly neutrophils and macrophages (Figure 6C). This strong correlation reinforces the connection between these hub genes and the recruitment or activation of key innate immune cells during the EIP.

### 3.7 Experimental validation in a murine AMI model confirms upregulation of hub Genes

**Figure 7:**
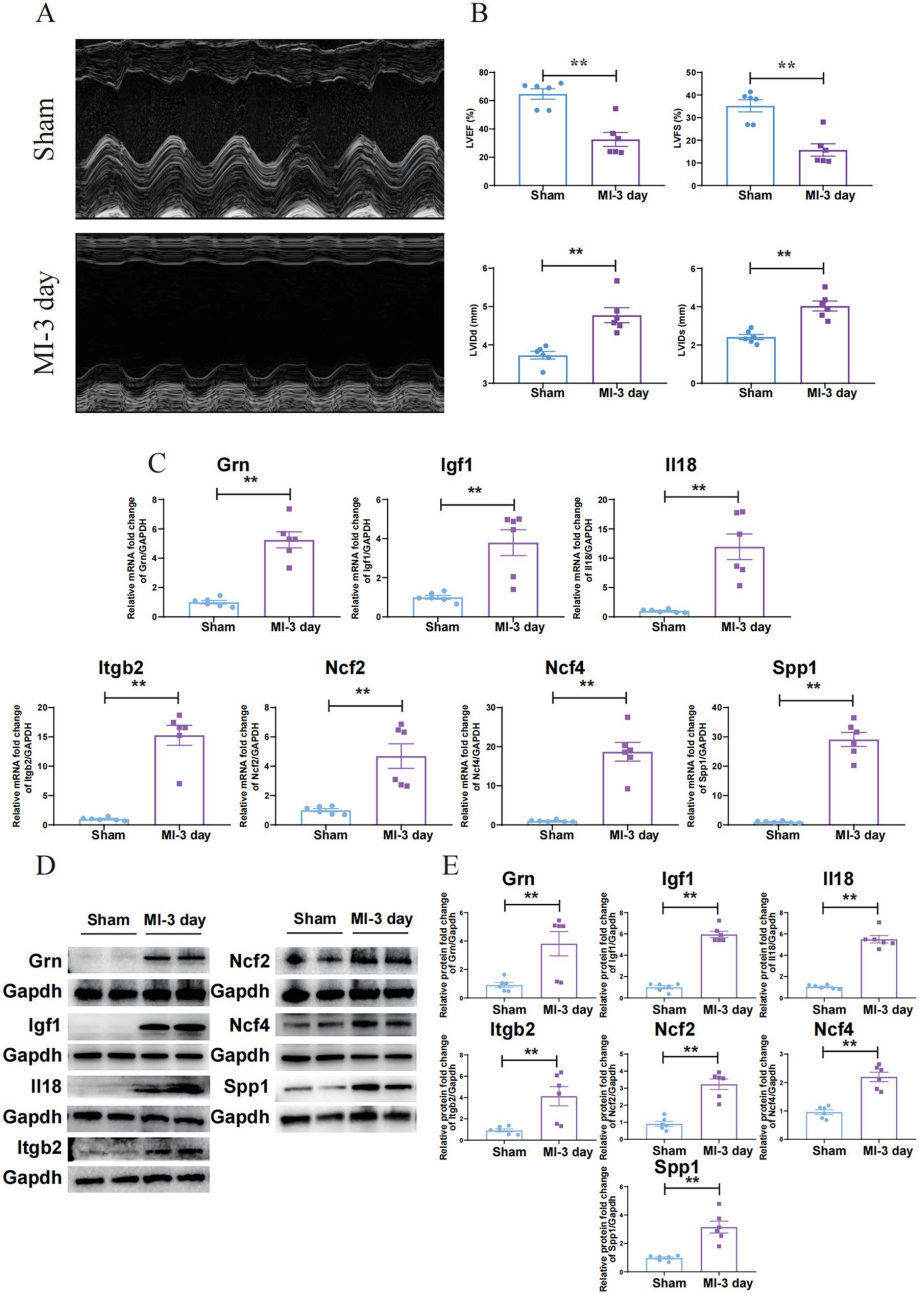
Experimental validation of hub gene expression in a murine AMI model. Representative M-mode echocardiographic images from Sham and MI mice at day 3 post-surgery. **(B)** Quantification of left ventricular functional parameters: ejection fraction (EF%), fractional shortening (FS%), left ventricular internal diameter at end-diastole and end-systole (LVIDd and LVIDs). *n* = 6 mice per group. **(C)** mRNA expression levels of the seven hub genes in the peri-infarct myocardium quantified by qRT-PCR and normalized to Gapdh. *n* = 6 mice per group. **(D,E)** Western blot analysis of hub protein expression in the peri-infarct myocardium. Representative immunoblots for the seven hub proteins and GAPDH (loading control) from two biological replicates per group are shown on the left. The corresponding densitometric quantification of all samples (*n* = 6 mice per group), normalized to GAPDH, is presented on the right. Data are mean ± SEM. **p < 0.01 (unpaired two-tailed Student's *t*-test or Mann–Whitney *U* test).

To validate the AMI model, cardiac function was assessed by echocardiography at day 3 post-surgery. Mice subjected to LAD ligation exhibited significantly reduced ejection fraction (EF) and fractional shortening (FS), along with increased left ventricular dimensions (LVIDd, LVIDs), compared to the Sham group (Figures 7A,B), confirming successful induction of cardiac dysfunction.

Consistent with this functional deficit and our bioinformatic predictions, the ventricular expression levels of the seven inflammatory hub genes were significantly upregulated at the molecular level. qRT-PCR analysis of peri-infarct tissue showed increased mRNA levels for *Grn, Igf1, Il18, Itgb2, Ncf2, Ncf4*, and *Spp1* in the MI group (Figure 7C). Western blot analysis further confirmed the upregulation of these hub genes at the protein level (Figure 7D and Supplementary Figure S8). Together, these data verify the successful establishment of the AMI model and confirm the *in vivo* relevance of the identified inflammatory network during the early phase.

## 4 Discussion

Despite major advances in percutaneous coronary intervention, nearly one-third of patients with acute myocardial infarction (AMI) still suffer from progressive cardiac dysfunction, which can ultimately lead to severe heart failure and death (25). Numerous studies have demonstrated that immuno-inflammatory processes in the early phase (within 3 days) post-MI play a decisive role in cardiac outcomes (4–6), yet understanding of the key regulatory molecules in this phase remains insufficient. Therefore, this study provides a multi-dimensional transcriptomic dissection of the critical early inflammatory phase (EIP) following AMI. By integrating temporal bulk RNA sequencing with single-cell transcriptome sequencing, we move beyond a static snapshot to capture the dynamic evolution of gene programs and immune cell interactions within the decisive 0–72 h window. Our work culminates in the identification of a core network of seven inflammatory hub genes (*Grn*, *Igf1*, *Il18*, *Itgb2*, *Ncf2*, *Ncf4*, *Spp1*) that are consistently upregulated, correlate with myeloid cell infiltration, and show promising diagnostic potential. These findings offer a refined framework for understanding the coordinated immune response that shapes early post-infarct remodeling.

The paramount importance of the EIP is increasingly recognized, as interventions in this narrow window can decisively influence the transition from injury to repair (3, 5–7). Our temporal trend analysis strongly supports this concept, revealing a phased yet overlapping activation of pro-inflammatory (e.g., leukocyte activation, chemokine signaling) and early reparative (e.g., extracellular matrix organization) pathways within 72 h. Subsequent single-cell sequencing illuminated the cellular drivers of these programs, showing a massive shift toward a myeloid-dominated immune landscape, a hallmark of the early injury response (26). Importantly, cell–cell communication analysis further revealed that this period is characterized not only by cellular influx but by significantly enhanced intercellular crosstalk, particularly through pathways such as FN1 and GALECTIN, which are known to amplify inflammatory signaling and recruitment (20, 21). This holistic view underscores the EIP as a period of intense and coordinated cellular activity, making it a critical target for therapeutic modulation.

Ischemic insults trigger a series of compensatory responses, from early inflammatory activation to subsequent healing (27). Maintaining a delicate pro-/anti-inflammatory balance is crucial for sustaining cardiac microenvironment homeostasis. The core of our study lies in the identification and validation of seven hub genes. It is important to consider whether the observed inflammatory signature is a primary driver of pathology or a secondary consequence of cell death. While initial cardiomyocyte necrosis is the inciting event, substantial evidence indicates that the subsequent sterile inflammatory response is an active, non-redundant process that significantly amplifies injury and dictates remodeling outcomes (26). The coordinated upregulation of these hub genes, their strong association with infiltrating immune cells, and their diagnostic performance collectively suggest they are integral components of this active pathogenic response, rather than passive bystanders.

The hub genes themselves represent a functionally diverse and interconnected set. Among them, we identified five pro-inflammatory genes: *Itgb2*, *Ncf2*, *Ncf4*, *Spp1*, and *Il18*. As a motility integrin, Itgb2 (integrin subunit beta 2) is involved in immune-related activities and is especially implicated in the mobilization or migration of leukocytes, including macrophages, neutrophils, and dendritic cells (28, 29). Additionally, Itgb2 has been reported as a risk factor for atherothrombotic events by mediating the early recruitment of macrophages to activated vascular endothelium, thereby aggravating inflammation (30, 31). Ncf2 (neutrophil cytosolic factor 2) and Ncf4 are key cytosolic regulatory subunits of the NADPH oxidase enzyme, both responsible for robust reactive oxygen species (ROS) burst production, resulting in deleterious effects in several diseases linked to dysregulated immuno-inflammatory signaling (32–35). Nevertheless, few studies have directly analyzed the relationship between Ncf2/Ncf4 and ischemic cardiac dysfunction. In this study, the pro-inflammatory effects of Ncf2/Ncf4 have been linked to dysregulation of the immune microenvironment in AMI and may represent potential therapeutic targets. Osteopontin (Spp1), a secreted glycophosphoprotein belonging to the cell-extracellular matrix family, has been reported to participate in inflammatory and reparative responses post-AMI, with higher expression in immune cells (36). Furthermore, the crucial role of Spp1 in cardiac remodeling has been attributed to its regulation of MMP activity and collagen deposition after AMI (37). Interleukin-18 (Il18) is a potent pro-inflammatory cytokine that links the NLRP3 inflammasome to the pathogenesis of cardiovascular diseases including atherosclerosis, myocarditis, and AMI (38). Therefore, the pathogenic roles of these five pro-inflammatory hub genes require further study to identify effective targets for intervening in early post-AMI inflammation.

Moreover, the activation of anti-inflammatory signals appears to be a crucial mediator of inflammatory homeostasis by preventing cytokine storms and modulating the inflammatory response (39). Therefore, a great need remains to identify novel targets for treating AMI by modulating the imbalance of pro-/anti-inflammatory immune activity during the EIP. Interestingly, we found that two hub genes possessed pronounced anti-inflammatory activity. Grn encodes a multifunctional growth factor, progranulin, which has been suggested to have anti-inflammatory effects due to its potential regulatory role in macrophage polarization and cytokine (e.g., IL-10, TNF) production (40–42). However, the correlation between Grn and inflammatory response-associated genes in the AMI immune microenvironment has not been reported. Similarly, the concentration of insulin-like growth factor 1 (Igf1) has been used as a marker of the severity and outcomes of coronary artery disease and is considered a potential cardiovascular risk factor (43, 44). Nederlof et al. found that Igf1 treatment confers an anti-inflammatory phenotype to neutrophils and macrophages, accompanied by downregulation of pro-inflammatory cytokines (TNF-α and IL6) (45). Their inclusion in this network likely reflects the concurrent activation of counter-regulatory mechanisms aimed at controlling inflammation and promoting repair. This duality suggests our signature captures the complex balance of the early response. From a translational perspective, this has important implications: the pro-inflammatory genes (*Itgb2*, *Ncf2*, *Ncf4*, *Spp1*, *Il18*) may represent more direct therapeutic targets for intervention, while the full signature, encapsulating both pro- and anti-inflammatory elements, may be more powerful as a diagnostic or prognostic biomarker panel reflecting the overall intensity and character of the EIP.

A pertinent question is why classic early cytokines like IL-1β or TNF-α were not among our final hub genes. These genes were differentially expressed in our datasets but did not rank among the top network nodes in our WGCNA-based screen. This may indicate that our analysis, focused on sustained co-expression patterns over the 72-h window, identified genes that function as stable regulatory or effector components within the inflammatory program, rather than earlier, more transient triggers. Our hub genes, such as the NADPH oxidase components, may be involved in executing and perpetuating the damaging effects initiated by those primary cytokines.

Our study has several limitations. First, while integrated, the scRNA-seq analysis is constrained by the limited biological replicates available in public datasets, a common challenge in such exploratory analyses. Second, the clinical exploratory analysis is limited by the small sample size (entire cohort: 26 AMI vs. 7 controls; STEMI subgroup: 7 vs. 7) and lack of an independent validation cohort. Therefore, the reported diagnostic AUCs should be interpreted as hypothesis-generating only; large-scale prospective studies with standardized sampling are urgently needed to validate the clinical utility of these genes. Third, and most critically, our work establishes strong associations but does not prove causality. The definitive mechanistic roles of these hub genes in post-MI inflammation and remodeling await rigorous functional validation through gain- and loss-of-function studies *in vitro* and *in vivo*. This is a key direction for future research. Fourth, the immune infiltration analysis is entirely computational and lacks experimental corroboration (e.g., by flow cytometry or immunohistochemistry). Fifth, histological validation of infarct size (e.g., TTC staining) and spatial protein-level analysis (e.g., immunohistochemistry for hub genes and immune markers) were not performed, constrained by the scope and resources of this primarily omics-driven discovery and validation study. Our AMI model was instead rigorously validated by functional echocardiography and the consistent upregulation of the identified molecular signature, which serve as direct indicators of cardiac injury and the ensuing inflammatory response. Future studies employing these spatial techniques will provide valuable complementary insights. Finally, while our multi-step bioinformatics pipeline is rigorous, any analytical approach carries inherent biases; we have provided our code as Supplementary File to ensure transparency.

In conclusion, by leveraging complementary transcriptomic approaches, we have delineated the dynamic gene expression and immune cell communication networks that define the early inflammatory phase of AMI. We have identified and validated a core set of seven hub genes that are central to this response. This work not only advances our molecular understanding of early post-infarct pathophysiology but also provides a valuable resource and specific, high-priority candidates for future investigations aimed at developing timing-specific diagnostic tools and immunomodulatory therapies to improve outcomes after AMI.

## Data Availability

The datasets presented in this study can be found in online repositories. The names of the repository/repositories and accession number(s) can be found below: RNA-seq data are available in the GEO database under accession numbers GSE206281, GSE153494, GSE163129, GSE163465, GSE161427, and GSE60993.
