# Supplementary material for: Integrated multi-omics profiling of the early post-infarct heart reveals a hub gene network associated with myeloid-driven inflammation
Source: Front Cardiovasc Med. 2026 Jul 13;13:1837094. doi: 10.3389/fcvm.2026.1837094 (PMC13402454; doi:10.3389/fcvm.2026.1837094)
Supplement: Supplementary file 1 [file Table1.docx]

Supplementary Material

# Supplementary Figures and Tables

| **Name** | **Primer** |
| --- | --- |
| Grn | F-5′ATGTGGGTCCTGATGAGCTG 3′ |
|  | R-5′GCTCGTTATTCTAGGCCATGTG 3′ |
| Igf1 | F-5′ CACATCATGTCGTCTTCACACC 3′ |
|  | R-5′GGAAGCAACACTCATCCACAATG 3′ |
| Il18 | F-5′ GACTCTTGCGTCAACTTCAAGG 3′ |
|  | R-5′ CAGGCTGTCTTTTGTCAACGA 3′ |
| Itgb2 | F-5′TGCCGCATTCAATGTGACTTT 3′ |
|  | R-5′CTTCTTGACGTTGTTGAGGTCAT 3′ |
| Ncf2 | F-5′ GCTGCGTGAACACTATCCTGG 3′ |
|  | R-5′ AGGTCGTACTTCTCCATTCTGTA 3′ |
| Ncf4 | F-5′ GTCATCGAGGTCAAAACAAAAGG 3′ |
|  | R-5′GCCCATGTAGACTTTGGCTG 3′ |
| Spp1 | F-5′AGAGCGGTGAGTCTAAGGAGT 3′ |
|  | R-5′ TGCCCTTTTCCGTTGTTGTCC 3′ |
| Gapdh | F-5′ TGGCCTTCCGTGTTCCTAC 3′ |
|  | R-5′ GAGTTGCTGTTGAAGTCGCA 3′ |

**Table 1.** Nucleotide sequences of primers for Grn, Igf1, Il18, Itgb2, Ncf2, Ncf4, Spp1, and Gapdh.


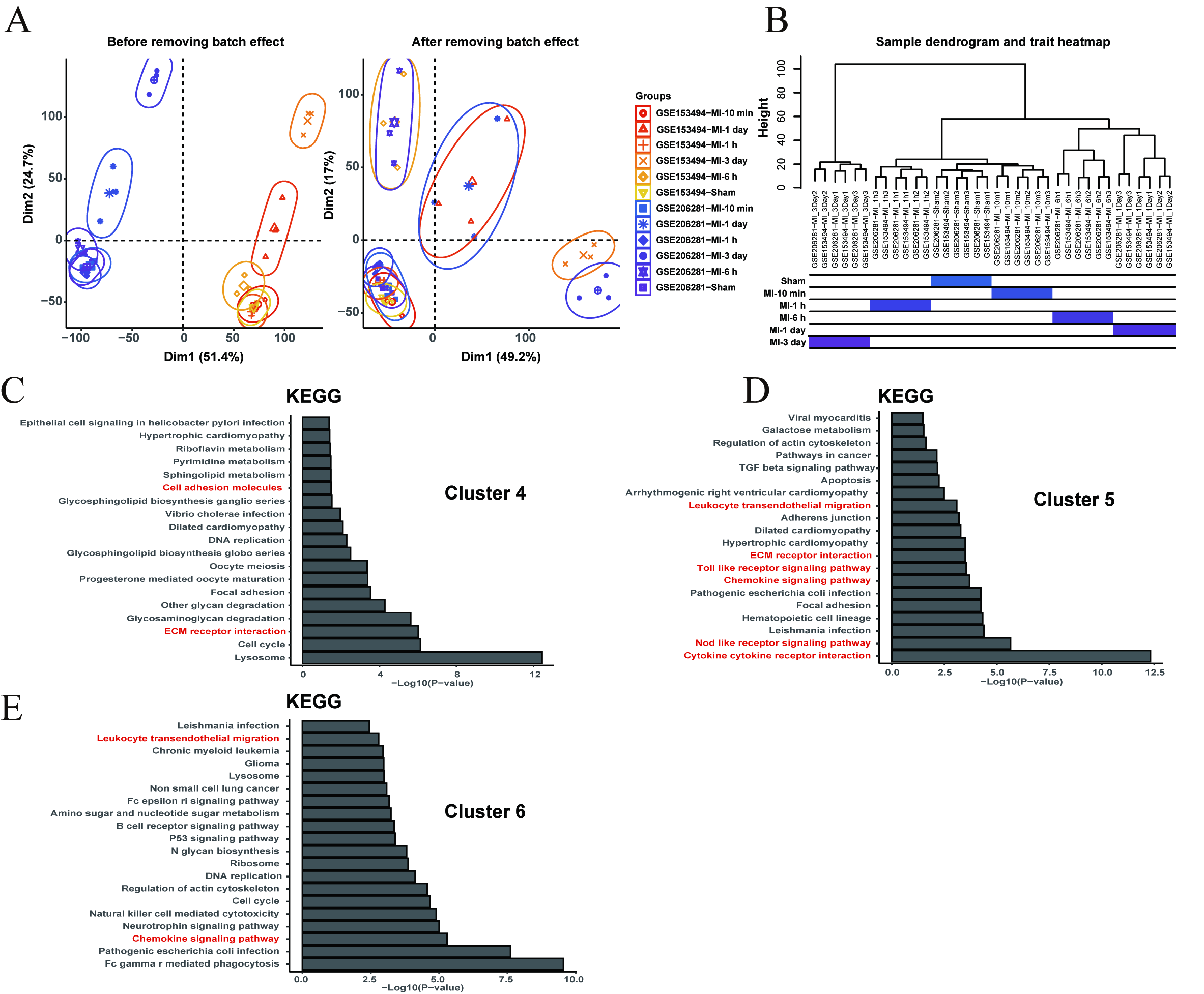


Figure S1. Quality control and pathway analysis for temporal transcriptomic data. (A) Principal component analysis (PCA) plot before and after batch effect correction (ComBat) for the GSE206281 and GSE153494 datasets. (B) Hierarchical clustering of all samples based on gene expression profiles. (C-E) The top enriched Kyoto Encyclopedia of Genes and Genomes (KEGG) pathways for Mfuzz clusters 4, 5, and 6, respectively.

**
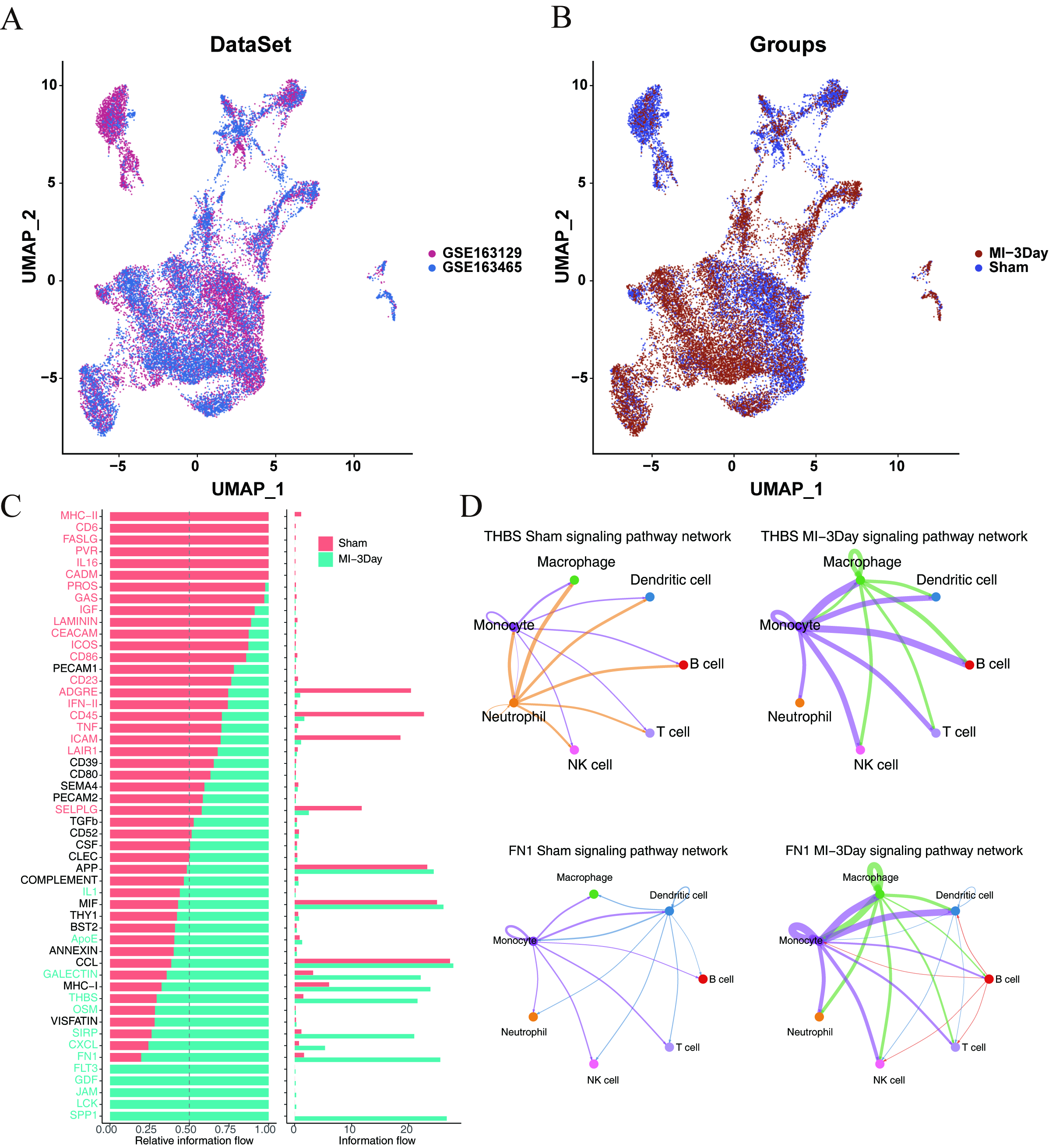
**

Figure S2. Extended analysis of cell-cell communication. (A-B) UMAP plots colored by original dataset source and experimental group, respectively, demonstrating successful integration. (C) Signaling pathways with significant differences in the overall information flow between Sham and MI-3day conditions. (D) Circle plots displaying the differential activity of the THBS (upper) and FN1 (lower) signaling pathways between groups.

**
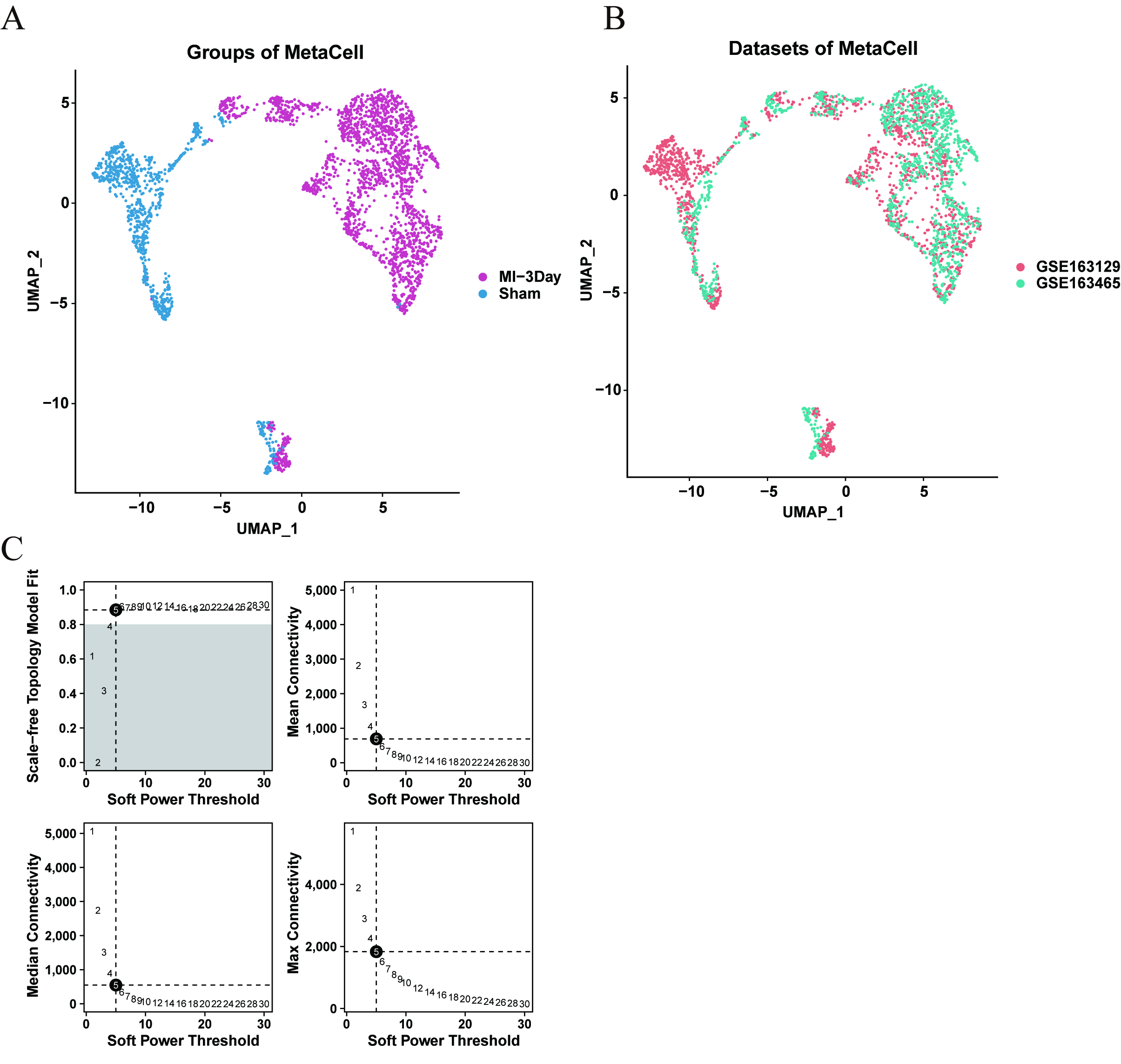
**

Figure S3. Metacell generation and WGCNA network construction. (A-B) UMAP visualization of the metacells generated from high-IPS myeloid subsets, colored by group and dataset. (C) Analysis of network topology for various soft-thresholding powers. Left: The scale-free fit index (R2). Right: The mean connectivity. The selected power is indicated.

**
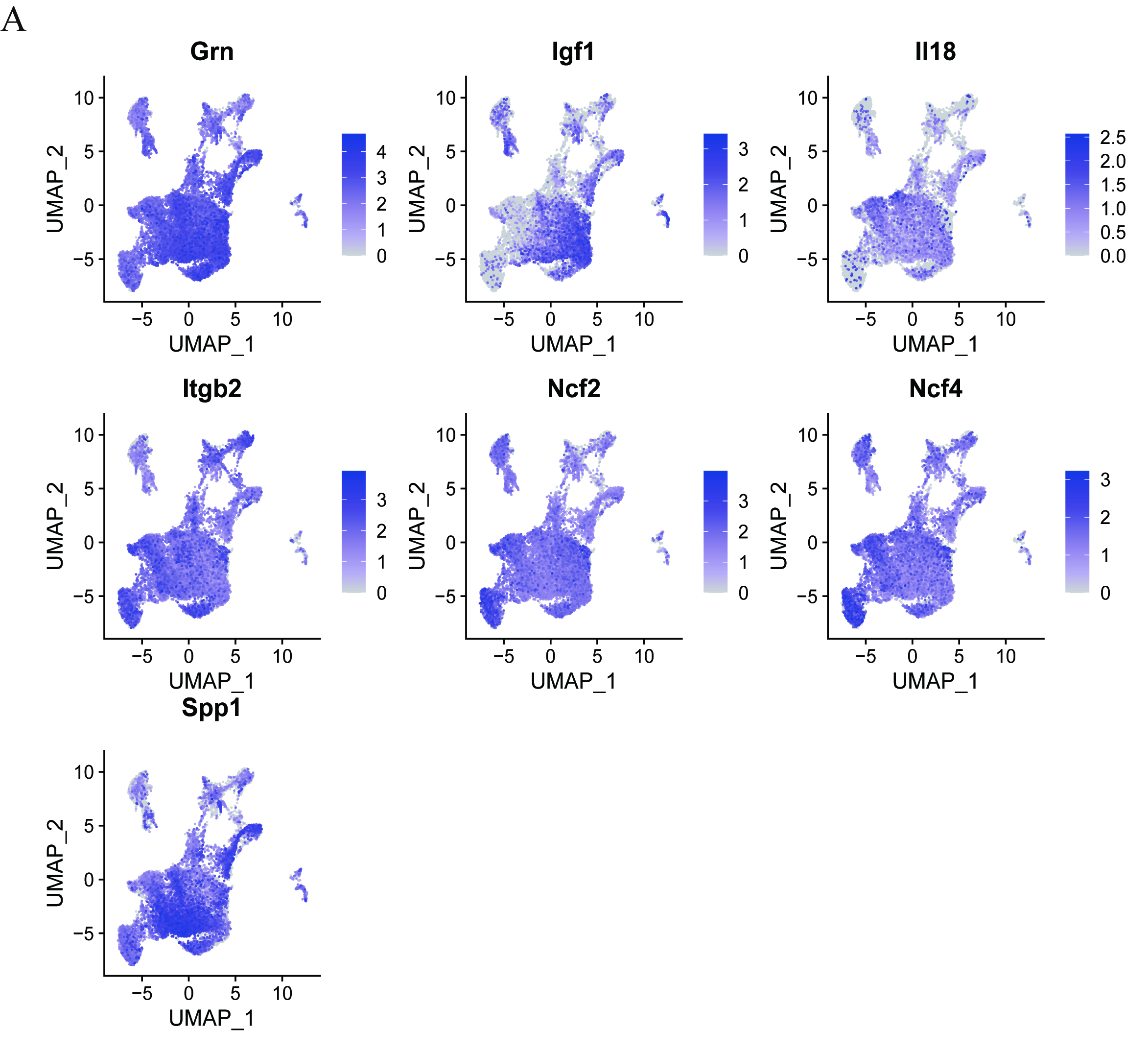
**

Figure S4. Spatial expression patterns of hub genes in the single-cell atlas. UMAP plots showing the expression and distribution of each of the seven hub genes (Grn, Igf1, Il18, Itgb2, Ncf2, Ncf4, Spp1) across the integrated single-cell dataset.

**
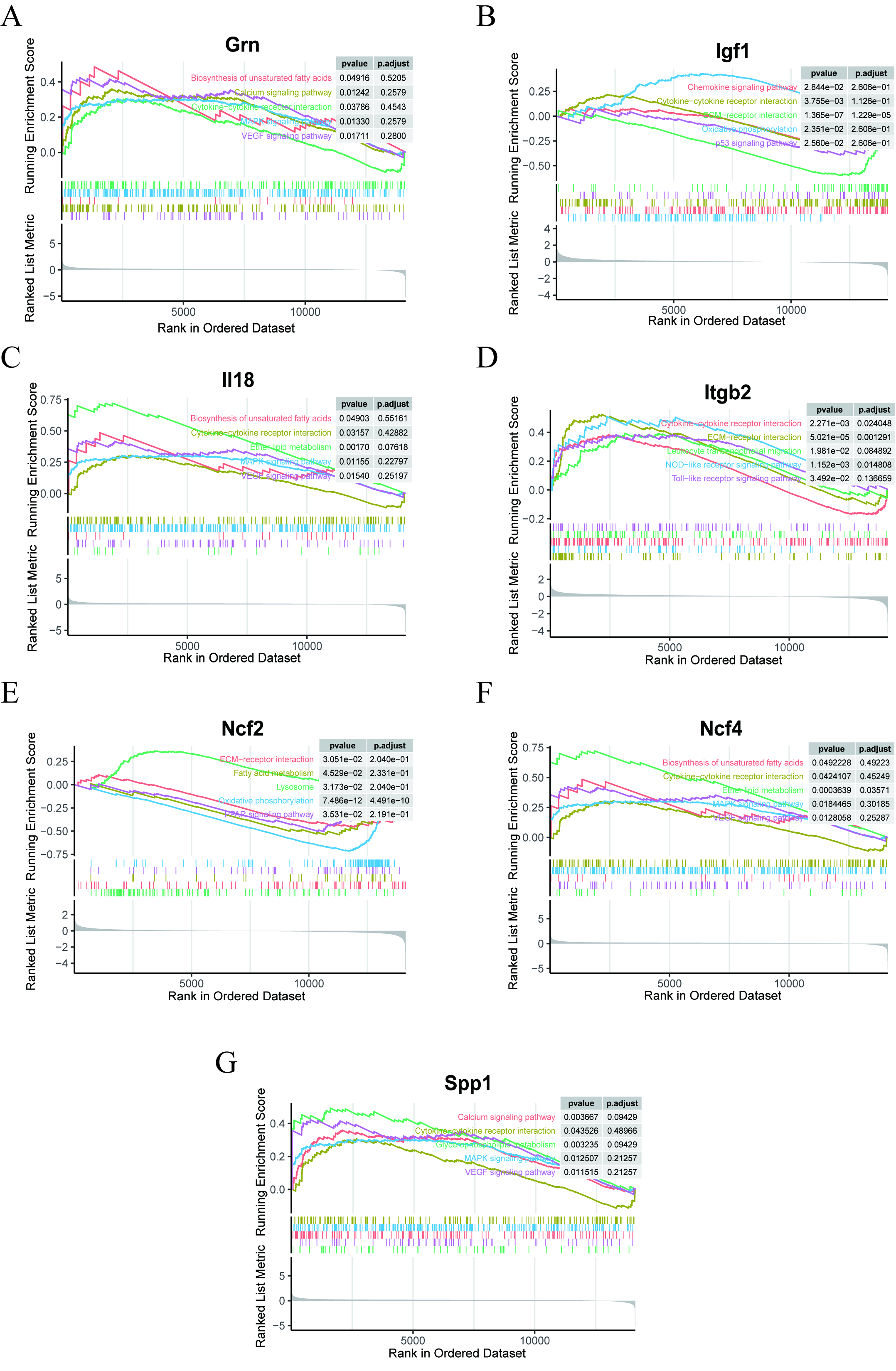
**

Figure S5. Gene Set Enrichment Analysis (GSEA) for hub gene signatures. Enrichment plots from GSEA showing significant enrichment of hallmark inflammatory and metabolic pathways (e.g., Inflammatory Response, Oxidative Phosphorylation) in samples with high expression signatures of the seven hub genes.

**
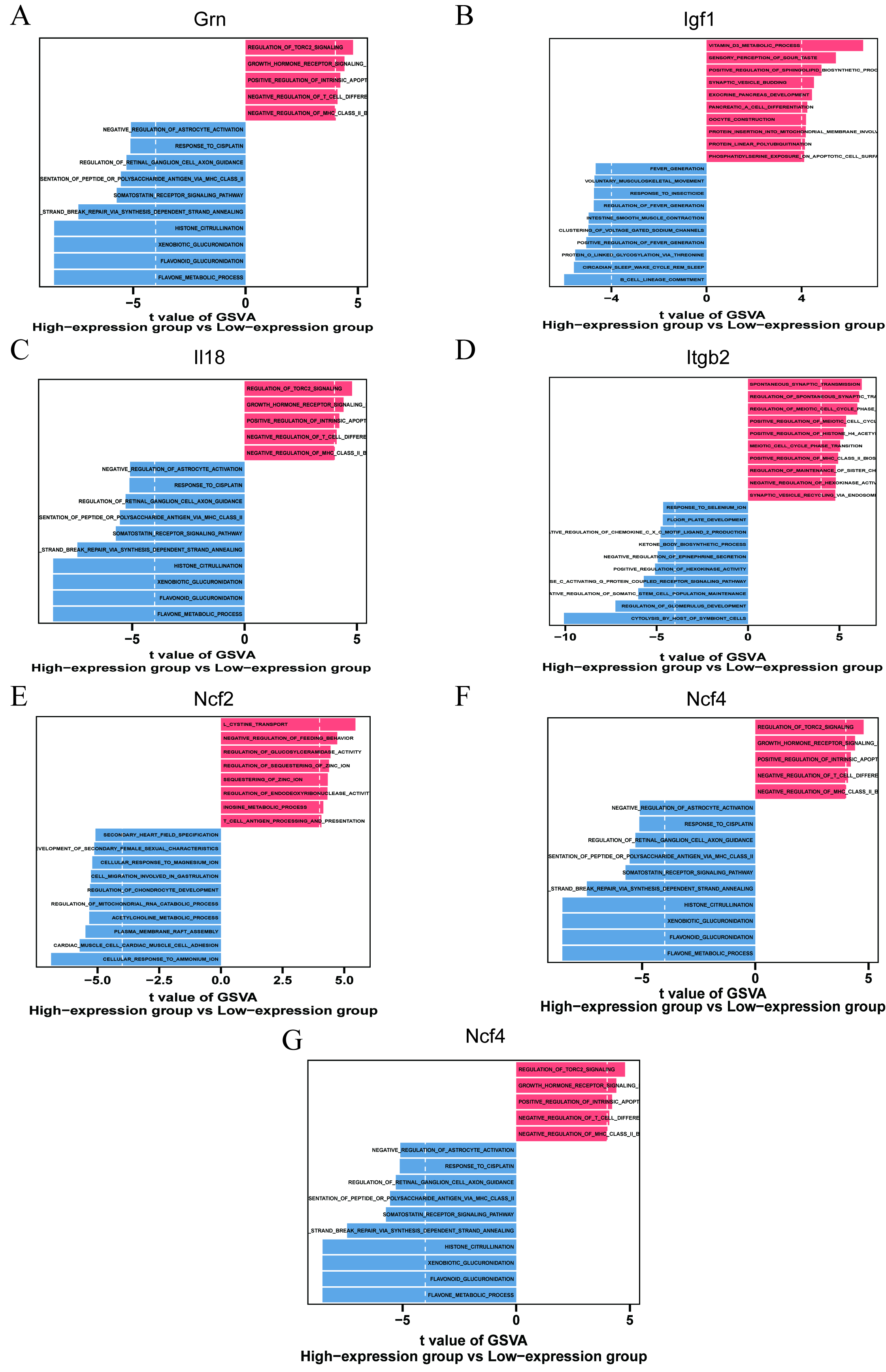
**

Figure S6. Gene Set Variation Analysis (GSVA) for pathway activity. Heatmap depicting the GSVA scores of selected KEGG pathways across samples, highlighting coordinated activation of immune and repair-related pathways in the MI-3day group.

**
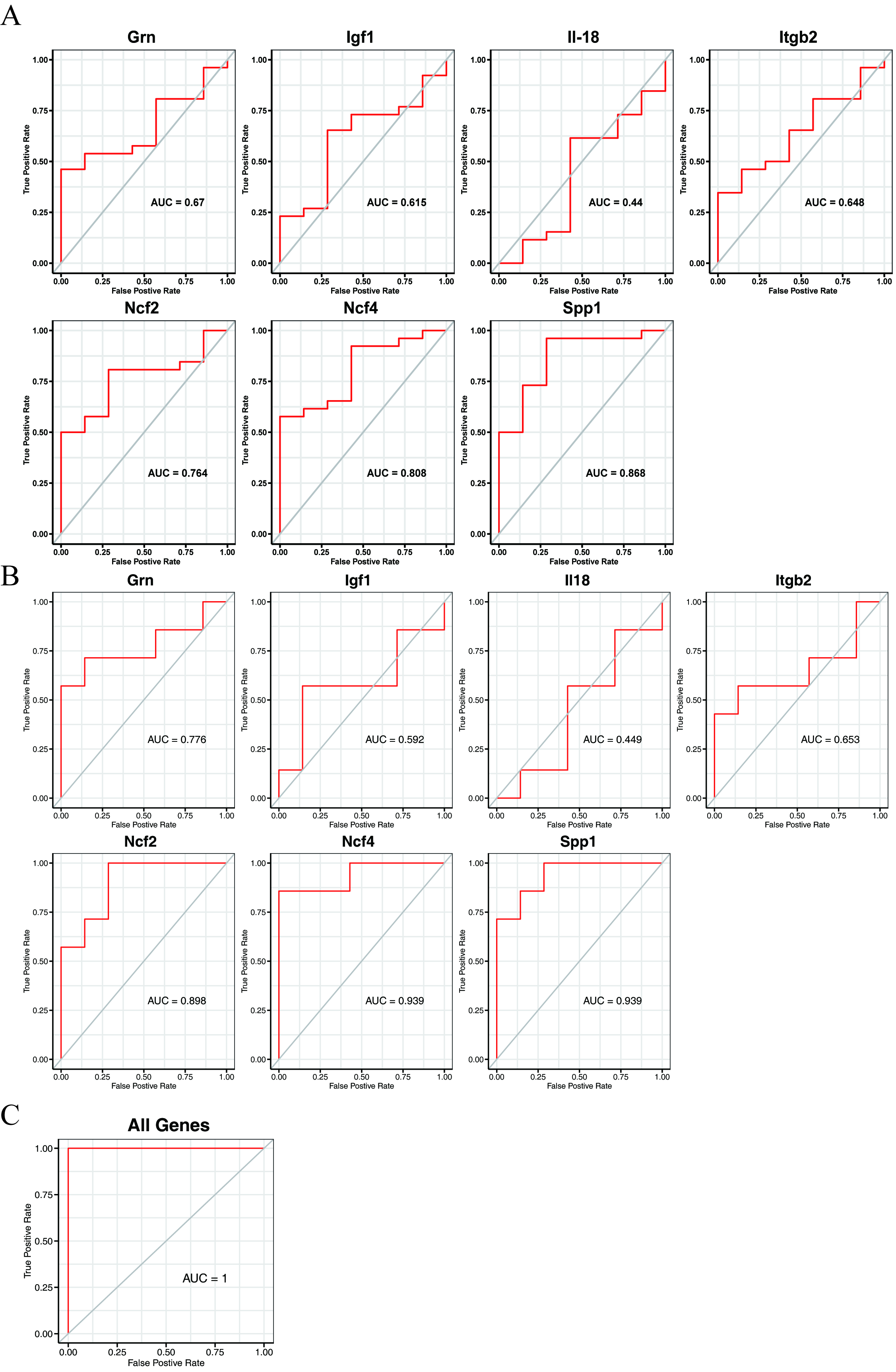
**

Figure S7. Detailed diagnostic performance of the hub gene signature. (A) ROC curves and individual AUC values for each of the seven hub genes in distinguishing AMI patients from controls in the GSE60993 cohort (all samples). (B) Individual gene ROC curves after excluding Non-ST-elevation MI (NSTEMI) and Unstable Angina (UA) samples, retaining only STEMI (n=18) and Control (n=10) subjects. (C) ROC curve for the composite seven-gene signature in the STEMI-only subgroup. Note: The perfect AUC (1.0) in this analysis is likely influenced by the limited sample size and highlights the need for validation in larger, independent cohorts.
