# Supplementary material for: Integrated multi-omics profiling of the early post-infarct heart reveals a hub gene network associated with myeloid-driven inflammation
Source: Front Cardiovasc Med. 2026 Jul 13;13:1837094. doi: 10.3389/fcvm.2026.1837094 (PMC13402454; doi:10.3389/fcvm.2026.1837094)
Supplement: Supplementary file 2 [file Table2.docx]

Part 1 Code for process.R

library(ggplot2)

library(Seurat)

library(data.table)

library(Seurat)

library(dplyr)

library(patchwork)

library(DoubletFinder)

library(randomcoloR)

library(momr)

library(pheatmap)

library(forcats)

library(ggstance)

library(clusterProfiler)

library(msigdbr)

library(enrichplot)

library(ggstatsplot)

library(Hmisc)

library(limma)

library(factoextra)

library(ggforce)

library(sva)

library(ggrepel)

library(ggplot2)

library(org.Mm.eg.db)

library(DOSE)

library(AUCell)

Find_doublet <- function(data){

sweep.res.list <- paramSweep_v3(data, PCs = 1:20, sct = FALSE)

sweep.stats <- summarizeSweep(sweep.res.list, GT = FALSE)

bcmvn <- find.pK(sweep.stats) ### output plot

nExp_poi <- round(0.05*ncol(data))

p <- as.numeric(as.vector(bcmvn[bcmvn$MeanBC==max(bcmvn$MeanBC),]$pK)) ### pK Selection

data <- doubletFinder_v3(data, PCs = 1:20, pN = 0.25, pK = p, nExp = nExp_poi, reuse.pANN = FALSE, sct = FALSE) ### output plot

colnames(data@meta.data)[ncol(data@meta.data)] = "doublet_info"

return(data)

}

setwd("E:/xianyu/chenliyuan")

barcodes <- read.table("GSE146285_barcodes_used.txt", sep = "\t", header = T, row.names = 1)

samples <- list.files(path = ".//GSE146285_RAW", full.names = F, recursive = F)

data_list <- c()

for (i in samples) {

print(i)

data <- fread(input = paste0(".//GSE146285_RAW//",i),

sep = "\t", header = F)

data <- as.data.frame(data)

data$V1 <- unlist(lapply(data$V1, function(x){

unlist(strsplit(x, "_"))[1]

}))

data <- data[!duplicated(data$V1),]

rownames(data) <- data$V1

colnames(data) <- data[1,]

data <- data[,-1]

data <- data[-1,]

colnames(data) <- barcodes[colnames(data),1]

sample_id <- paste(unlist(strsplit(i, "_"))[1:3], collapse = "-")

colnames(data) <- paste(colnames(data), sample_id, sep = "-")

data <- CreateSeuratObject(data, project = sample_id)

data_list <- c(data_list, list(data))

}

seu <- merge(x = data_list[[1]], y = list(data_list[[2]],

data_list[[3]],

data_list[[4]],

data_list[[5]],

data_list[[6]],

data_list[[7]],

data_list[[8]],

data_list[[9]]))

rm(data_list)

seu$Group <- unlist(lapply(seu$orig.ident, function(x){

paste(unlist(strsplit(x, "-"))[2:3], collapse = "-")

}))

seu$Sample <- unlist(lapply(seu$orig.ident, function(x){

unlist(strsplit(x, "-"))[1]

}))

seu[["percent.mt"]] <- PercentageFeatureSet(seu, pattern = "^mt-")

nFeature_RNA <- VlnPlot(seu, features = c("nFeature_RNA")) +

theme(axis.text.x = element_text(angle = 90, hjust = 1, vjust = 0.5)) +

xlab("") +

NoLegend()

nCount_RNA <- VlnPlot(seu, features = c("nCount_RNA")) +

theme(axis.text.x = element_text(angle = 90, hjust = 1, vjust = 0.5)) +

xlab("") +

NoLegend()

percent.mt <- VlnPlot(seu, features = c("percent.mt")) +

theme(axis.text.x = element_text(angle = 90, hjust = 1, vjust = 0.5)) +

xlab("") +

NoLegend()

pdf("SI_1_QC.pdf", width = 9, height = 6)

cowplot::plot_grid(nFeature_RNA, nCount_RNA, percent.mt, ncol = 3)

dev.off()

seu <- subset(seu, subset = nCount_RNA > 1000 & nFeature_RNA > 200 & percent.mt < 25)

data_list <- SplitObject(seu, split.by = "ident")

for (i in 1:length(data_list)) {

print(i)

temp <- data_list[[i]]

temp <- NormalizeData(object = temp, verbose = FALSE)

temp <- FindVariableFeatures(object = temp)

temp <- ScaleData(temp)

temp <- RunPCA(temp, verbose=F)

temp <- RunUMAP(temp, dims = 1:20)

temp <- Find_doublet(temp)

data_list[[i]] <- temp

}

seu <- merge(x = data_list[[1]], y = list(data_list[[2]],

data_list[[3]],

data_list[[4]],

data_list[[5]],

data_list[[6]],

data_list[[7]],

data_list[[8]],

data_list[[9]]))

Idents(seu) <- seu$doublet_info

seu <- NormalizeData(object = seu, verbose = FALSE)

seu <- FindVariableFeatures(object = seu)

seu <- ScaleData(seu)

seu <- RunPCA(seu, verbose=F)

seu <- RunUMAP(seu, dims = 1:20)

pdf("Figure1_1.pdf", height = 5, width = 5+max(nchar(seu$doublet_info))*0.1)

DimPlot(seu, cols = c("black","red")) +

ggtitle(label = "Doublets") + theme(plot.title = element_text(hjust = 0.5))

dev.off() #

seu <- subset(seu, subset = doublet_info == "Singlet")

seu <- NormalizeData(object = seu, verbose = T)

seu <- FindVariableFeatures(object = seu)

seu <- ScaleData(seu)

seu <- RunPCA(seu, verbose=F)

seu <- JackStraw(seu, num.replicate = 100, dims = 50)

seu <- ScoreJackStraw(seu, dims = 1:50)

pdf("SI_2_JackStrawPlot.pdf", width = 9.3, height = 6)

JackStrawPlot(seu, dims = 1:25)

dev.off()

seu <- RunUMAP(seu, dims = 1:20)

seu <- RunTSNE(seu, dims = 1:20)

seu <- FindNeighbors(seu, dims = 1:20)

seu <- FindClusters(seu, resolution = 0.5)

# clusters_color <- randomColor(count = 11, hue = c(" ", "random", "red", "orange", "yellow",

# "green", "blue", "purple", "pink", "monochrome"), luminosity = "bright")

clusters_color <- c("#DD6AF2", "#FC882F", "#D64273", "#4272BF", "#AF3F2D",

"#F2E55C", "#29B58F", "#5054B7", "#419613", "#4218CC",

"#530984")

seu <- RenameIdents(seu,

"0" = "1",

"1" = "2",

"2" = "3",

"3" = "4",

"4" = "5",

"5" = "6",

"6" = "7",

"7" = "8",

"8" = "9",

"9" = "10",

"10" = "11")

seu$Clusters <- as.character(Idents(seu))

pdf("Figure1_2.pdf", height = 5, width = 5)

DimPlot(seu, cols = clusters_color, label = T, repel = T, label.box = T, label.color = "white") +

theme_bw() +

NoLegend() +

theme(panel.grid = element_blank())

dev.off()

pdf("SI_3_clusters_markers.pdf", width = 6, height = 6)

DotPlot(seu, features = c("Ttn", "Myh6", "Des", "Actc1", # Cardiomyocytes

"Col1a1", "Col3a1", "Fbln2", "Sparc", # Fibroblasts

"Pecam1", "Fabp4", "Tie1", "Egfl7", # Endothelial_cells

"Il1b", "S100a8", "Mmp9", "S100a9", # Immune_cells

"Cd68", "Lyz1", "Lgals3", "Itgam")) + # Macrophages

theme(axis.text.x = element_text(angle = 45, hjust = 1, vjust = 1)) +

labs(x = "", y = "Clusters") +

theme(legend.position = "top")

dev.off()

Idents(seu) <- seu$Clusters

seu <- RenameIdents(seu,

"1" = "1 Fibroblasts",

"2" = "2 Fibroblasts",

"3" = "3 Cardiomyocytes",

"4" = "4 Cardiomyocytes",

"5" = "5 Macrophages/Immune_cells",

"6" = "6 Endothelial_cells",

"7" = "7 Fibroblasts",

"8" = "8 Immune_cells",

"9" = "9 Cardiomyocytes",

"10" = "10 Endothelial_cells",

"11" = "11 Fibroblasts")

pdf("Figure1_2_legend.pdf", width = 4, height = 5)

DimPlot(seu, cols = clusters_color)

dev.off()

seu$Clustes_cellType <- Idents(seu)

seu$cellType <- unlist(lapply(as.character(seu$Clustes_cellType), function(x){

unlist(strsplit(x, " ", fixed = T))[2]

}))

Idents(seu) <- seu$cellType

cellType_marker <- FindAllMarkers(seu, only.pos = T, avg_log2FC.threshold = 0.25)

cellType_marker <- cellType_marker[order(cellType_marker$cluster, cellType_marker$avg_log2FC, decreasing = T),]

cellType_marker %>%

group_by(cluster) %>%

top_n(n = 10, wt = avg_log2FC) -> top10

exp <- seu@assays$RNA@data[as.character(top10$gene),]

exp <- as.data.frame(as.matrix(exp))

exp <- t(exp)

meta <- seu@meta.data

meta <- as.data.frame(cbind(celltype = meta$cellType,

as.data.frame(exp)))

top10 <- top10[,c(6,7)]

meta <- aggregate.data.frame(meta[,2:ncol(meta)],

by = list(meta$celltype),

FUN = mean)

rownames(meta) <- meta$Group.1

meta <- meta[,-1]

meta <- as.data.frame(t(meta))

meta <- meta[,levels(top10$cluster)]

anno_row <- data.frame(

celltype = factor(

rep(levels(top10$cluster), each = 10),

levels = levels(top10$cluster)

)

)

rownames(anno_row) <- rownames(meta)

anno_cor <- list(

celltype = c("Cardiomyocytes"="#9983BD", "Fibroblasts"="#D24B27",

"Endothelial_cells"="#3BBCA8", "Macrophages/Immune_cells"="#6E4B9E",

"Immune_cells"="#0C727C"),

font = "italic"

)

pdf("Figure1_3.pdf", width = 6, height = 12)

pheatmap(meta, cluster_rows = F, cluster_cols = F, annotation_row = anno_row, annotation_colors = anno_cor,

cellwidth = 22, cellheight = 10, scale = "row",

border_color = NA)

dev.off()

pdf("Figure1_4.pdf", height = 5, width = 5+2)

DimPlot(seu, group.by = "orig.ident") +

theme_bw() +

# NoLegend() +

theme(panel.grid = element_blank()) +

ggtitle("Samples") +

theme(plot.title = element_text(hjust = 0.5, face = "bold"))

dev.off()

meta <- seu@meta.data

meta_sham <- meta[which(meta$Group == "Sham-1day"),]

meta_sham <- as.data.frame(table(meta_sham$cellType))

meta_sham$Freq <- meta_sham$Freq / sum(meta_sham$Freq)

meta_sham$Freq <- round(meta_sham$Freq, 3)

meta_sham$color <- c("#9983BD","#3BBCA8","#D24B27","#0C727C","#6E4B9E")

meta_sham$Percent <- paste(meta_sham$Freq * 100, "%", sep = "")

meta_MI <- meta[which(meta$Group == "MI-3day"),]

meta_MI <- as.data.frame(table(meta_MI$cellType))

meta_MI$Freq <- meta_MI$Freq / sum(meta_MI$Freq)

meta_MI$Freq <- round(meta_MI$Freq, 3)

meta_MI$color <- c("#9983BD","#3BBCA8","#D24B27","#0C727C","#6E4B9E")

meta_MI$Percent <- paste(meta_MI$Freq * 100, "%", sep = "")

pdf("Figure1_5.pdf", width = 6, height = 3)

par(mfrow=c(1,2))

pie(meta_MI$Freq,

labels = paste(meta_MI$Var1, meta_MI$Percent, sep = "\n"),

col = meta_MI$color,

radius = 1, main = "MI 3 Day")

pie(meta_sham$Freq,

labels = paste(meta_sham$Var1, meta_sham$Percent, sep = "\n"),

col = meta_sham$color,

radius = 1, main = "Sham 1 Day")

dev.off()

meta <- seu@meta.data

meta <- as.data.frame(table(meta$Group, meta$cellType))

for (i in c("MI-3day", "Sham-1day")) {

meta[which(meta$Var1 == i),"Freq"] <- meta[which(meta$Var1 == i),"Freq"] / sum(meta[which(meta$Var1 == i),"Freq"])

}

meta$Var2 <- factor(as.character(meta$Var2),

levels = rev(c("Cardiomyocytes", "Endothelial_cells",

"Fibroblasts", "Macrophages", "Immune_cells")))

pdf("Figure1_6.pdf", width = 6, height = 3.5)

ggplot() +

geom_bar(data = meta, aes(x = Var2, y = Freq, fill = Var1),

stat = "identity", position = position_dodge2(), color = "black") +

theme_classic() +

coord_flip() +

theme(axis.text = element_text(size = 12),

axis.title = element_text(size = 12, face = "bold"),

legend.position=c(.85, .15)) +

labs(x = "", y = "Proportion of Cells", fill = "") +

scale_fill_manual(values = c("MI-3day" = "#E05197", "Sham-1day" = "#1E99D7"))

dev.off()

####### Figure2 #######

Idents(seu) <- seu$cellType

sub_seu <- subset(seu, idents = c("Macrophages", "Immune_cells"))

Idents(sub_seu) <- sub_seu$Group

all_single_diff <- FindMarkers(sub_seu, ident.1 = "MI-3day", ident.2 = "Sham-1day", avg_log2FC.threshold = -Inf, min.pct = 0.01)

all_single_diff <- all_single_diff[,c(2,5)]

all_single_diff <- all_single_diff[order(all_single_diff$avg_log2FC,decreasing=T),]

write.csv(all_single_diff, "Figure2_singleCell_ALL_MI_vs_Sham.csv", quote = FALSE)

DOWN_row <- intersect(which(all_single_diff$p_val_adj < 0.05), which(all_single_diff$avg_log2FC < -1))

UP_row <- intersect(which(all_single_diff$p_val_adj < 0.05), which(all_single_diff$avg_log2FC > 1))

DOWN <- all_single_diff[DOWN_row,]

UP <- all_single_diff[UP_row,]

write.csv(DOWN, "Figure2_singleCell_DOWN_MI_vs_Sham.csv", quote = FALSE)

write.csv(UP, "Figure2_singleCell_UP_MI_vs_Sham.csv", quote = FALSE)

DOWN <- DOWN[order(DOWN$avg_log2FC,decreasing = F),]

UP <- UP[order(UP$avg_log2FC,decreasing = T),]

sigdiff <- as.data.frame(rbind(UP[1:13,],

DOWN[1:8,]))

color <- rep("gray", length(all_single_diff$avg_log2FC))

cex <- rep(1.2, length(all_single_diff$avg_log2FC))

cex[DOWN_row] <- 2

cex[UP_row] <- 2

cex <- as.character(cex)

color[DOWN_row] <- "blue"

color[UP_row] <- "red"

pdf("Figure2_1.pdf", width = 6.5, height = 5)

vio_plot <- ggplot(data = all_single_diff, aes(x = avg_log2FC, y = -log10(p_val_adj), size = cex, colour = color, alpha = "0.3")) +

theme_bw() +

labs(x = "Log2 FoldChange", y = "log10 Adjusted P value") +

geom_point() +

geom_hline(aes(yintercept=-log10(0.05)), colour = "black", linetype="dashed") +

geom_vline(aes(xintercept= -1), colour="black", linetype="dashed") +

geom_vline(aes(xintercept= 1), colour="black", linetype="dashed") +

scale_color_manual(name = "",

values = c('red' = 'red', "blue" = 'blue', "gray" = "gray"),

labels = c("\nUP(Log2FC > 1, Adjusted P < 0.05)\nN = 307\n", '\nDOWN(Log2FC < -1, Adjusted P < 0.05)\nN = 53\n', "Not significant")) +

scale_size_manual(values = c('1.2' = 1.2, "2" = 2)) +

scale_alpha_manual(values = c("0.3" = 0.3, "1" = 1)) +

guides(size = "none", alpha = "none")

vio_plot +

geom_text_repel(data = sigdiff, aes(x = avg_log2FC,

y = -log10(p_val_adj),

label = rownames(sigdiff),

alpha = rep("1",nrow(sigdiff))),

size = 3,

box.padding = unit(0.8, "lines"),

point.padding = unit(0, "lines"),

min.segment.length = 0,

segment.color = "black",

colour="#000000",

show.legend = FALSE,

max.overlaps = getOption("ggrepel.max.overlaps", default = 30))

dev.off()

exp <- seu@assays$RNA@data[c(rownames(DOWN),rownames(UP)),]

exp <- as.data.frame(as.matrix(exp))

exp <- t(exp)

meta <- seu@meta.data

meta <- as.data.frame(cbind(Samples = meta$orig.ident,

as.data.frame(exp)))

meta <- aggregate.data.frame(meta[,2:ncol(meta)],

by = list(meta$Samples),

FUN = mean)

rownames(meta) <- meta$Group.1

meta <- meta[,-1]

meta <- as.data.frame(t(meta))

meta <- as.data.frame(t(meta))

for (i in 1:ncol(meta)) {

meta[,i] <- (meta[,i] - mean(meta[,i])) / sd(meta[,i])

}

meta <- as.data.frame(t(meta))

max(meta)

min(meta)

anno_row <- data.frame(

DEG_type = factor(

c(rep("DOWN",nrow(DOWN)),rep("UP",nrow(UP))),

levels = c("UP", "DOWN")

)

)

rownames(anno_row) <- rownames(meta)

anno_col <- data.frame(

Group = factor(

c(rep("Sham",4),rep("MI-3day",5)),

levels = c("Sham", "MI-3day")

)

)

rownames(anno_col) <- colnames(meta)

anno_cor <- list(

DEG_type = c("DOWN" = "blue", "UP" = "red"),

Group = c("Sham" = "#1E99D7",

"MI-3day" = "#E05197")

)

meta[meta >= 1.5] <- 1.5

meta[meta <= -1.5] <- -1.5

pdf("Figure2_2.pdf", width = 5, height = 9)

pheatmap(meta, cluster_rows = T, cluster_cols = T, annotation_row = anno_row,

annotation_colors = anno_cor, annotation_col = anno_col,

border_color = NA, show_rownames = F)

dev.off()

C5 <- msigdbr(species = "Mus musculus", category = "C5")

C5 <- C5[which(C5$gs_subcat %in% c("GO:BP", "GO:CC", "GO:MF")),]

diff_list <- all_single_diff$avg_log2FC

names(diff_list) <- rownames(all_single_diff)

GO_gsea <- GSEA(diff_list, TERM2GENE = C5[,c(3,4)], verbose = T, eps = 0)

GO_gsea_result <- as.data.frame(GO_gsea@result)

GO_gsea_result$Description <- unlist(lapply(GO_gsea_result$Description, function(x){

paste(tolower(unlist(strsplit(x,"_"))[-1]), collapse = " ")

}))

GO_gsea_result$Description <- capitalize(GO_gsea_result$Description)

GO_gsea_result$Ontology <- unlist(lapply(GO_gsea_result$ID, function(x){

unlist(strsplit( unlist(strsplit(x,"_"))[1], "GO"))[2]

}))

GO_gsea_result <- GO_gsea_result[order(GO_gsea_result$Ontology, -GO_gsea_result$NES),]

write.csv(GO_gsea_result, "Figure2_singleCell_GO_gsea_result.csv")

saveRDS(seu, "SingleCellSeurat.rds")

############

# Idents(seu) <- seu$cellType

# Cardiomyocytes <- subset(seu, idents = "Cardiomyocytes")

# Cardiomyocytes <- NormalizeData(object = Cardiomyocytes, verbose = FALSE)

# Cardiomyocytes <- FindVariableFeatures(object = Cardiomyocytes)

# Cardiomyocytes <- ScaleData(Cardiomyocytes)

# Cardiomyocytes <- RunPCA(Cardiomyocytes, verbose=F)

# Cardiomyocytes <- RunUMAP(Cardiomyocytes, dims = 1:20)

# DimPlot(Cardiomyocytes, group.by = "Group")

library(TxDb.Mmusculus.UCSC.mm10.knownGene)

########### bulk 3d ###########

GSE206281_IDMAP <- read.csv("./bulk_3d/GSE206281_IDMAP.txt", sep = "\t", header = T, row.names = 1)

GSE206281 <- read.csv("./bulk_3d/GSE206281_FPKM_allsamples.csv", header = T, row.names = 1, check.names = F)

GSE206281 <- data.frame(Symbol = GSE206281_IDMAP[rownames(GSE206281),1],

GSE206281)

GSE206281 <- GSE206281[-which(GSE206281$Symbol == ""),]

GSE206281 <- aggregate.data.frame(GSE206281[,2:ncol(GSE206281)], by = list(GSE206281$Symbol), FUN = mean)

rownames(GSE206281) <- GSE206281$Group.1

GSE206281 <- GSE206281[,-1]

colnames(GSE206281) <- c("GSE206281-Sham1", "GSE206281-Sham2", "GSE206281-Sham3",

"GSE206281-MI_10m1", "GSE206281-MI_10m2", "GSE206281-MI_10m3",

"GSE206281-MI_1h1", "GSE206281-MI_1h2", "GSE206281-MI_1h3",

"GSE206281-MI_6h1", "GSE206281-MI_6h2", "GSE206281-MI_6h3",

"GSE206281-MI_1Day1", "GSE206281-MI_1Day2", "GSE206281-MI_1Day3",

"GSE206281-MI_3Day1", "GSE206281-MI_3Day2", "GSE206281-MI_3Day3")

GSE153494 <- read.csv("./bulk_3d/GSE153494_All_sample_FPKM.csv", header = T, row.names = 1, check.names = F)

colnames(GSE153494) <- c("GSE153494-Sham1", "GSE153494-Sham2", "GSE153494-Sham3",

"GSE153494-MI_10m1", "GSE153494-MI_10m2", "GSE153494-MI_10m3",

"GSE153494-MI_1h1", "GSE153494-MI_1h2", "GSE153494-MI_1h3",

"GSE153494-MI_6h1", "GSE153494-MI_6h2", "GSE153494-MI_6h3",

"GSE153494-MI_1Day1", "GSE153494-MI_1Day2", "GSE153494-MI_1Day3",

"GSE153494-MI_3Day1", "GSE153494-MI_3Day2", "GSE153494-MI_3Day3")

GSE206281 <- log2(GSE206281 + 1)

GSE153494 <- log2(GSE153494 + 1)

keep_num <- c()

for (i in 1:nrow(GSE206281)) {

temp <- GSE206281[i,] == 0

sample_0 <- sum(temp[1,])

# Sham_0 <- sum(temp[1,1:3])

# MI_0 <- sum(temp[1,4:6])

if (sample_0 <= 3) {

keep_num <- c(keep_num, i)

}

}

GSE206281 <- GSE206281[keep_num,]

keep_num <- c()

for (i in 1:nrow(GSE153494)) {

temp <- GSE153494[i,] == 0

sample_0 <- sum(temp[1,])

# Sham_0 <- sum(temp[1,1:3])

# MI_0 <- sum(temp[1,4:6])

if (sample_0 <= 3) {

keep_num <- c(keep_num, i)

}

}

GSE153494 <- GSE153494[keep_num,]

common_genes <- intersect(rownames(GSE206281),rownames(GSE153494))

mergeData <- as.data.frame(cbind(

GSE206281[common_genes,],

GSE153494[common_genes,]

))

group <- as.factor(c(rep("GSE206281-Sham", 3),

rep("GSE206281-MI_10m", 3),

rep("GSE206281-MI_1h", 3),

rep("GSE206281-MI_6h", 3),

rep("GSE206281-MI_1day", 3),

rep("GSE206281-MI_3day", 3),

rep("GSE153494-Sham", 3),

rep("GSE153494-MI_10m", 3),

rep("GSE153494-MI_1h", 3),

rep("GSE153494-MI_6h", 3),

rep("GSE153494-MI_1day", 3),

rep("GSE153494-MI_3day", 3)

))

pca_before <- prcomp(as.data.frame(t(mergeData)), scale = TRUE)

P1 <- fviz_pca_ind(pca_before, label = "none",

habillage = group, addEllipses = FALSE,

palette = c(heat.colors(10)[2:7], colorRampPalette(c("#4169E1", "purple"))(6))) +

theme_classic() +

theme(plot.title = element_text(hjust = 0.5, face = "bold"),

legend.position = "none") +

ggtitle(label = "Before removing batch effect") +

ggforce::geom_mark_ellipse(aes(color = group))

# mod <- as.factor(c(rep("Sham", 3), rep("MI", 3), rep("Sham", 3), rep("MI", 3)))

# mod <- model.matrix(~mod)

batch <- factor(c(rep("GSE206281", 18),

rep("GSE153494", 18)))

combat_data <- ComBat(dat = mergeData, batch = batch)

combat_data[which(combat_data < 0)] <- 0

combat_data <- as.data.frame(combat_data)

pca_after_combat <- prcomp(as.data.frame(t(combat_data)), scale = TRUE)

P2 <- fviz_pca_ind(pca_after_combat, label = "none",

habillage = group, addEllipses = FALSE,

palette = c(heat.colors(10)[2:7], colorRampPalette(c("#4169E1", "purple"))(6))) +

theme_classic() +

theme(plot.title = element_text(hjust = 0.5, face = "bold")) +

ggtitle(label = "After removing batch effect") +

ggforce::geom_mark_ellipse(aes(color = group))

P1 + P2

pdf("Figure2_3.pdf", height = 5, width = 11)

P1 + P2

dev.off()

saveRDS(combat_data, "After_remove_batchEffect.rds")

#### Mfuzz

library(Mfuzz)

Matrix <- as.data.frame(t(combat_data)) # 行是sample，列是gene

traitData <- data.frame(Group = c(rep("Sham", 3), rep("MI_10m", 3), rep("MI_1h", 3), rep("MI_6h", 3), rep("MI_1day", 3), rep("MI_3day", 3),

rep("Sham", 3), rep("MI_10m", 3), rep("MI_1h", 3), rep("MI_6h", 3), rep("MI_1day", 3), rep("MI_3day", 3)),

Sample = rownames(Matrix),

row.names = rownames(Matrix))

traitData$Group <- factor(traitData$Group, levels = c("Sham", "MI_10m", "MI_1h", "MI_6h", "MI_1day", "MI_3day"))

traitData <- traitData[order(traitData$Group),]

Matrix <- data.frame(Group = traitData[rownames(Matrix),"Group"],

Matrix)

Matrix <- aggregate.data.frame(Matrix[,2:ncol(Matrix)], by = list(Matrix$Group), FUN = mean)

rownames(Matrix) <- Matrix[,1]

Matrix <- Matrix[,-1]

Matrix <- as.data.frame(t(Matrix))

mfuzz_class <- new('ExpressionSet', exprs = as.matrix(Matrix))

#预处理缺失值或者异常值

mfuzz_class <- filter.NA(mfuzz_class, thres = 0.25)

mfuzz_class <- fill.NA(mfuzz_class, mode = 'mean')

mfuzz_class <- filter.std(mfuzz_class, min.std = 0.5)

mfuzz_class <- standardise(mfuzz_class)

set.seed(123)

cluster_num <- 6

mfuzz_cluster <- mfuzz(mfuzz_class, c = cluster_num, m = mestimate(mfuzz_class))

# pdf("Figure2_4.pdf", width = 10.5, height = 7)

mfuzz.plot2(mfuzz_class, cl = mfuzz_cluster, mfrow = c(2, 3),

time.labels = colnames(Matrix), centre = TRUE)

mfuzzColorBar(main="Membership Value",cex.main=1)

# dev.off()

#查看每个聚类群中各自包含的基因数量

cluster_size <- mfuzz_cluster$size

names(cluster_size) <- 1:cluster_num

cluster_size

# 查看每个基因所属的聚类群

head(mfuzz_cluster$cluster)

# Mfuzz 通过计算一个叫 membership 的统计量判断基因所属的聚类群，以最大的 membership 值为准

# 查看各基因的 membership 值

head(mfuzz_cluster$membership)

#最后，提取所有基因所属的聚类群，并和它们的原始表达值整合在一起

gene_cluster <- mfuzz_cluster$cluster

gene_cluster <- cbind(Matrix[names(gene_cluster), ], gene_cluster)

head(gene_cluster)

write.table(gene_cluster, 'Figure2_gene_cluster.txt', sep = '\t', col.names = NA, quote = FALSE)

##########

## cluster genes enrichment

library(Hmisc)

C5 <- msigdbr(species = "Mus musculus", category = "C5")

table(C5$gs_subcat)

C5 <- C5[C5$gs_subcat%in%c("GO:BP","GO:CC","GO:MF"),]

msigdbr_GO <- C5 %>% dplyr::select(gs_name, gene_symbol) %>% as.data.frame()

C2 <- msigdbr(species = "Mus musculus", category = "C2")

table(C2$gs_subcat)

C2 <- C2[C2$gs_subcat%in%c("CP:KEGG"),]

msigdbr_KEGG <- C2 %>% dplyr::select(gs_name, gene_symbol) %>% as.data.frame()

for (i in unique(gene_cluster$gene_cluster)) {

temp_cluster_gene <- rownames(gene_cluster)[which(gene_cluster$gene_cluster == i)]

KEGG <- enricher(gene = temp_cluster_gene, TERM2GENE = msigdbr_KEGG, pvalueCutoff = 1, qvalueCutoff = 1)

KEGG_result <- as.data.frame(KEGG)

KEGG_result <- KEGG_result[which(KEGG_result$pvalue < 0.05),]

KEGG_result$Description <- unlist(lapply(KEGG_result$Description, function(x){

temp <- unlist(strsplit(x, "_"))[-1]

temp <- paste(temp, collapse = " ")

}))

KEGG_result$Description <- tolower(KEGG_result$Description)

KEGG_result$Description <- capitalize(KEGG_result$Description)

KEGG@result <- KEGG_result

write.table(KEGG_result, paste0("./第二部分/cluster_genes_",i,"_KEGG_result.xls"), sep = "\t", quote = F, row.names = F)

KEGG_result$Description <- factor(KEGG_result$Description, levels = KEGG_result$Description)

KEGG_result <- KEGG_result[1:20,]

KEGG_result <- as.data.frame(na.omit(KEGG_result))

pdf(paste0("./第二部分/cluster_genes_",i,"_KEGG_result_top20.pdf"), height = 0.3*nrow(KEGG_result), width = max(nchar(as.character(KEGG_result[,"Description"])))*0.14+0.5)

p <- ggplot() +

geom_bar(data = KEGG_result, aes(x = Description, y = -log10(pvalue)), color = "black", stat = "identity") +

theme_classic() +

coord_flip() +

labs(x = "", y = "-Log10(P-value)") +

theme(axis.text = element_text(size = 10))

print(p)

dev.off()

}

for (i in unique(gene_cluster$gene_cluster)) {

temp_cluster_gene <- rownames(gene_cluster)[which(gene_cluster$gene_cluster == i)]

GO <- enricher(gene = temp_cluster_gene, TERM2GENE = msigdbr_GO, pvalueCutoff = 1, qvalueCutoff = 1)

GO_result <- as.data.frame(GO)

GO_result <- GO_result[which(GO_result$pvalue < 0.05),]

GO_result$ONCOLOGY <- unlist(lapply(GO_result$ID, function(x){

unlist(strsplit(x, "_"))[1]

}))

GO_result$Description <- unlist(lapply(GO_result$Description, function(x){

temp <- unlist(strsplit(x, "_"))[-1]

temp <- paste(temp, collapse = " ")

}))

GO_result$Description <- tolower(GO_result$Description)

GO_result$Description <- capitalize(GO_result$Description)

GO@result <- GO_result

write.table(GO_result, paste0("./第二部分/cluster_genes_",i,"_GO_result.xls"), sep = "\t", quote = F, row.names = F)

GO_result$Description <- factor(GO_result$Description, levels = GO_result$Description)

pdf(paste0("./第二部分/cluster_genes_",i,"_GO_result_top20.pdf"), height = 6, width = max(nchar(as.character(GO_result[1:20,"Description"])))*0.14+0.5)

p <- ggplot() +

geom_bar(data = GO_result[1:20,], aes(x = Description, y = -log10(pvalue), fill = ONCOLOGY), color = "black", stat = "identity") +

theme_classic() +

coord_flip() +

labs(fill = "Ontology", x = "", y = "-Log10(P-value)") +

theme(axis.text = element_text(size = 10))

print(p)

dev.off()

}

##########

cluster3 <- rownames(gene_cluster)[which(gene_cluster$gene_cluster == 3)]

cluster4 <- rownames(gene_cluster)[which(gene_cluster$gene_cluster == 4)]

cluster6 <- rownames(gene_cluster)[which(gene_cluster$gene_cluster == 6)]

up_genes <- rownames(UP)

####

library(gplots)

library(VennDiagram)

Tvenn <- venn.diagram(list(Mfuzz_C3=cluster3,Mfuzz_C4=cluster4,Mfuzz_C6=cluster6,MI_3day_UP=up_genes),

filename=NULL,

lwd=1,lty=2,

col=c('black','black','black','black') ,

fill=c('green','blue','purple','red'),

cat.col=c('black','black','black','black'),

reverse=TRUE)

pdf("Figure2_5.pdf", width = 5, height = 4.5)

grid.draw(Tvenn)

dev.off()

common_genes <- c(intersect(cluster3, up_genes),

intersect(cluster4, up_genes),

intersect(cluster6, up_genes))

common_genes_diff <- UP[common_genes,]

common_genes_diff <- common_genes_diff[order(common_genes_diff$avg_log2FC, decreasing = T),]

common_genes_diff$order <- 1:nrow(common_genes_diff)

common_genes_diff$Symbol <- rownames(common_genes_diff)

common_genes_diff$Clusters <- "Cluster3"

common_genes_diff[which(common_genes_diff$Symbol %in% cluster4),"Clusters"] <- "Cluster4"

common_genes_diff[which(common_genes_diff$Symbol %in% cluster6),"Clusters"] <- "Cluster6"

common_genes_diff$Symbol <- paste(common_genes_diff$Symbol,

common_genes_diff$Clusters,

sep = "_")

write.csv(common_genes_diff, "Figure2_6_data.csv", quote = F)

# 168 target genes function enrichment

library(msigdbr)

library(clusterProfiler)

library(org.Mm.eg.db)

library(DOSE)

C5 <- msigdbr(species = "Mus musculus", category = "C5")

table(C5$gs_subcat)

C5 <- C5[C5$gs_subcat%in%c("GO:BP","GO:CC","GO:MF"),]

msigdbr_GO <- C5 %>% dplyr::select(gs_name, gene_symbol) %>% as.data.frame()

GO <- enricher(gene = rownames(common_genes_diff), TERM2GENE = msigdbr_GO, pvalueCutoff = 1,qvalueCutoff = 1)

GO_result <- as.data.frame(GO@result)

GO_result <- GO_result[which(GO_result$pvalue < 0.05),]

# GO_result$ID <- factor(GO_result$ID, levels = rev(GO_result$ID))

GO_result$Description <- unlist(lapply(GO_result$Description, function(x){

temp <- unlist(strsplit(x, "_"))[-1]

paste(temp, collapse = " ")

}))

GO_result$Description <- tolower(GO_result$Description)

GO_result$Description <- capitalize(GO_result$Description)

GO_result$ONTOLOGY <- unlist(lapply(GO_result$ID, function(x){

temp <- unlist(strsplit(x, "_"))[1]

}))

# GO_result$Description <- factor(GO_result$Description, levels = rev(GO_result$Description))

GO_result <- GO_result[order(GO_result$ONTOLOGY,

GO_result$pvalue),]

top50 <- GO_result %>% group_by(ONTOLOGY) %>% slice_head(n = 50)

write.table(top50, "./168genes_GO_result_top50.csv",

sep = ",", quote = F, row.names = F, col.names = T)

write.table(GO_result, "./168genes_GO_result_all.csv",

sep = ",", quote = F, row.names = F, col.names = T)

selected <- read.csv("168genes_selected_enriched_GO.txt",

sep = "\t", header = F)

GO_network <- c()

for (i in 1:nrow(selected)) {

temp_gene <- selected[i, "V8"]

temp_gene <- unlist(strsplit(temp_gene, "/", fixed = T))

temp <- data.frame(GO = selected[i,"V2"],

genes = temp_gene)

GO_network <- as.data.frame(rbind(GO_network,

temp))

}

write.table(GO_network, "GO_network.txt", sep = "\t", quote = F, row.names = F, col.names = T)

GO_network_infor <- data.frame(node = c(unique(GO_network$GO), unique(GO_network$genes)),

infor = c(rep("GO", 29),

rep("gene", 71)))

write.table(GO_network_infor, "GO_network_infor.txt", sep = "\t", quote = F, row.names = F, col.names = T)

GO_select <- GO

GO_select@result <- GO_select@result[selected$V1,]

GO_select@result$ID <- unlist(lapply(GO_select@result$ID, function(x){

temp <- unlist(strsplit(x, "_"))[1]

temp2 <- paste(unlist(strsplit(x, "_"))[-1], collapse = " ")

temp2 <- tolower(temp2)

temp2 <- Hmisc::capitalize(temp2)

paste(temp, temp2, sep = " | ")

}))

rownames(GO_select@result) <- GO_select@result$ID

GO_select@result$Description <- GO_select@result$ID

GO_select@result$Description <- factor(GO_select@result$Description,

levels = GO_select@result$Description)

pdf("new.GO_select_barplot.pdf", width = 9, height = 8)

barplot(GO_select, color = "pvalue", showCategory = 29, label_format = 100)

dev.off()

pdf("Figure2_6.pdf", height = 5, width = 5.8)

ggplot() +

geom_point(data = common_genes_diff,

aes(x = order, y = avg_log2FC, color = Clusters), alpha = 0.4, size = 2.5) +

scale_color_manual(values = c("Cluster3" = "green", "Cluster4" = "blue", "Cluster6" = "purple")) +

theme_bw() +

theme(panel.grid = element_blank(),

axis.text = element_text(size = 10),

axis.title = element_text(size = 13)) +

labs(x = "Rank Order", y = "Log2(FoldChange)") +

geom_text_repel(data = common_genes_diff[1:10,], aes(x = order,

y = avg_log2FC,

label = Symbol),

size = 3.5,

color = "red",

box.padding = unit(0.8, "lines"),

point.padding = unit(0, "lines"),

min.segment.length = 0,

segment.color = "black",

show.legend = FALSE,

max.overlaps = getOption("ggrepel.max.overlaps", default = 30))

dev.off()

##### 打分

seu <- AddModuleScore(seu, features = list(MI_genes = common_genes), search = TRUE)

pdf("Figure2_7.pdf", width = 5.5, height = 5)

FeaturePlot(seu, features = "Cluster1", reduction = "umap", cols = c("gray", "red")) +

ggtitle("MI Progression Score") +

theme(plot.title = element_text(hjust = 0.5, face = "bold"))

dev.off()

genes <- list(genes = unique(GO_network$genes))

cells_rankings <- AUCell_buildRankings(seu@assays$RNA@counts, nCores=1, plotStats=TRUE, splitByBlocks=TRUE)

cells_AUC <- AUCell_calcAUC(genes, cells_rankings)

cells_assignment <- AUCell_exploreThresholds(cells_AUC, plotHist=TRUE, assign=TRUE)

pdf("new.AUCell_score_thresholds.pdf", width = 4.5, height = 4.5)

AUCell_plotHist(cells_AUC["genes",], aucThr = 0.55)

abline(v = 0.55)

dev.off()

seu$AUCell_score <- cells_AUC@assays@data@listData[["AUC"]][1,colnames(seu)]

pdf("new.AUCell_score.pdf")

FeaturePlot(seu, features = "AUCell_score") +

scale_color_gradientn(values = seq(0, 1, 0.1),

colours = c('#333366',"#6666FF",'#CC3333','#FFCC33')) +

theme_bw() + theme(plot.title = element_text(hjust = 0.5, face = "bold"),

panel.grid.major = element_blank(),

panel.grid.minor = element_blank(), axis.line = element_line(colour = "black"),

panel.border = element_rect(fill=NA,color="black", size=0.7, linetype="solid"))

dev.off()

score_data <- seu@meta.data[,c("Clustes_cellType", "AUCell_score")]

p <- ggplot() +

geom_violin(data = score_data, aes(x = Clustes_cellType, y = AUCell_score, fill = Clustes_cellType),

trim = TRUE, color = "black") +

theme_classic() +

theme(axis.text.x = element_text(angle = 45, hjust = 1, vjust = 1),

legend.position = "none") +

scale_fill_manual(values = clusters_color) +

labs(x = "", y = "AUCell Score") +

geom_boxplot(data = score_data, aes(x = Clustes_cellType, y = AUCell_score),

width = 0.1, outlier.shape = NA) +

theme(axis.text = element_text(size = 10),

axis.title = element_text(size = 13))

pdf("new.cluster_celltype_AUCell_score.pdf", height = 4.5, width = 5)

p

dev.off()

seu$AUCell_status <- ifelse(seu$AUCell_score > 0.55, "ON", "OFF")

pdf("new.AUCell_ON_OFF.pdf", width = 4.5, height = 4.5)

DimPlot(seu, group.by = "AUCell_status", cols = c("gray", "#FF6347")) +

ggtitle("AUC > 0.55") +

theme_bw() + theme(plot.title = element_text(hjust = 0.5, face = "bold"),

panel.grid.major = element_blank(),

panel.grid.minor = element_blank(), axis.line = element_line(colour = "black"),

panel.border = element_rect(fill=NA,color="black", size=0.7, linetype="solid")) +

NoLegend()

dev.off()

data <- data.frame(table(seu$Clustes_cellType,

seu$AUCell_status))

pdf("new.ON_OFF_percent.pdf", width = 4.5, height = 5)

ggplot(data, aes(x = Var1, y = Freq, fill = Var2)) +

geom_bar(stat = "identity", position = "fill") +

labs(x = "", y = "Percent", fill = "Activity Binary") +

theme_bw() + theme(plot.title = element_text(hjust = 0.5, face = "bold"),

panel.grid.major = element_blank(),

panel.grid.minor = element_blank(), axis.line = element_line(colour = "black"),

panel.border = element_rect(fill=NA,color="black", size=0.7, linetype="solid")) +

theme(axis.text.x = element_text(angle = 90, vjust = 0.5, hjust = 1),

axis.text = element_text(size = 10),

axis.title.y = element_text(size = 12)) +

scale_fill_manual(values = c("OFF" = "gray", "ON" = "#FF6347"))

dev.off()

###### 细胞浸润

saveRDS(combat_data, "combat_data.rds")

setwd("../ImmucellAI")

combat_data <- readRDS("../combat_data.rds")

combat_data_immu <- combat_data[,c("GSE206281-Sham1","GSE206281-Sham2","GSE206281-Sham3",

"GSE153494-Sham1","GSE153494-Sham2","GSE153494-Sham3",

"GSE206281-MI_3Day1","GSE206281-MI_3Day2","GSE206281-MI_3Day3",

"GSE153494-MI_3Day1","GSE153494-MI_3Day2","GSE153494-MI_3Day3")]

combat_data_immu <- 2^combat_data_immu - 1

combat_data_immu <- as.data.frame(rbind(Group = c(rep("group1",6),rep("group2",6)),

combat_data_immu))

combat_data_immu <- as.data.frame(cbind(ID = rownames(combat_data_immu),

combat_data_immu))

write.table(combat_data_immu, "combat_data_immu.txt", sep = "\t", row.names = F, col.names = T, quote = F)

abundance_cell <- read.table("ImmuCellAI_mouse_abundance_result.txt",

sep = "\t", header = T, row.names = 1)

abundance_cell <- abundance_cell[,c(8:36)]

abundance_cell <- as.matrix(abundance_cell)

abundance_cell <- t(abundance_cell)

#整理成为ggplot2适用的格式

library(ggplot2)

ggplot_input <- data.frame()

for (i in 1:ncol(abundance_cell)) {

for (j in 1:nrow(abundance_cell)) {

ggplot_input <- rbind(ggplot_input, cbind(sample = colnames(abundance_cell)[i],

cell = rownames(abundance_cell)[j],

proportion = abundance_cell[j,i]))

}

}

ggplot_input[,3] <- as.data.frame(as.numeric(ggplot_input[,3]))

ggplot_input$sample <- factor(ggplot_input$sample,

levels = unique(ggplot_input$sample))

pdf("Figure3_1.pdf", width = 7, height = 7)

barstack <- ggplot(data=ggplot_input, aes(x=sample, y=proportion, fill=cell)) +

geom_col(position = "fill", color = "black")+ xlab("Samples") +

ylab("Immune cells infiltration ratio (%)") +

theme_classic() +

scale_y_continuous(expand = c(0.001,0.02)) +

theme(axis.text.x = element_text(angle = 45, hjust = 1, vjust = 1),

legend.title = element_text(face = "bold", size = 12),

axis.title.x.bottom = element_text(face = "bold", size = 12),

axis.title.y.left = element_text(face = "bold", size = 12)) +

theme(panel.grid.major = element_line(colour=NA)) +

labs(fill = "Celltypes")

barstack

dev.off()

library(pheatmap)

library(RColorBrewer)

library(ComplexHeatmap)

color <- colorRampPalette(rev(brewer.pal(n = 7, name = "RdYlBu")))(100)

anno_col <- data.frame(

Group = factor(rep(c("Sham","MI_3Day"), c(6,6)))

)

rownames(anno_col) <- colnames(abundance_cell)

anno_cor <- list(

Group = c(MI_3Day = "#FA8072", Sham = "#87CEFA")

)

abundance_cell <- t(abundance_cell)

imm_diff <- c()

for (i in 1:ncol(abundance_cell)) {

fc <- mean(abundance_cell[7:12,i]) / mean(abundance_cell[1:6,i])

p <- wilcox.test(abundance_cell[7:12,i], abundance_cell[1:6,i])

p <- p$p.value

imm_diff <- as.data.frame(rbind(imm_diff,

data.frame(Cell_type = colnames(abundance_cell)[i],

fc = fc,

p = p)))

}

fc <- imm_diff$fc

names(fc) <- imm_diff$Cell_type

color2 <- ifelse(imm_diff$fc > 1 & imm_diff$p < 0.05, "Significantly increased infiltration", NA)

abundance_cell <- t(abundance_cell)

pdf("Figure3_2.pdf", width = 7.5, height = 7.5)

p1 <- pheatmap(abundance_cell, color = color, cluster_rows = T, cluster_cols = F, name = "Proportion",

show_colnames = T, fontsize_row = 12, annotation_col = anno_col, border_color = NA,

annotation_colors = anno_cor, show_rownames = T, cellwidth = 10, cellheight = 14,

right_annotation = rowAnnotation(FC = anno_barplot(fc), FC_Type = color2, col = list(FC_Type = c("Significantly increased infiltration" = "red"))),

na_col = "white")

p1

dev.off()

gglist <- c()

for (i in c("M1_macrophage", "M2_macrophage", "Eosinophil", "Neutrophils")) {

temp_immu <- ggplot_input[which(ggplot_input$cell == i),]

temp_immu$Group <- anno_col[temp_immu$sample, 1]

p <- ggplot() +

geom_violin(data = temp_immu, aes(x = Group, y = proportion, color = Group, fill = Group),

trim = F, alpha = 0.2) +

geom_boxplot(data = temp_immu, aes(x = Group, y = proportion, fill = Group),

width = 0.2, color = "black", varwidth = T) +

geom_jitter(data = temp_immu, aes(x = Group, y = proportion, color = Group),

width = 0.15, shape = 18, size = 2, alpha = 0.7) +

labs(y = "Infiltration Proportion", x = "") +

theme_bw() +

ggtitle(i) +

theme(# panel.grid = element_blank(),

axis.text.x = element_text(angle = 45, hjust = 1, vjust = 1, face = "bold"),

legend.title = element_text(face = "bold", size = 12),

axis.title.x.bottom = element_text(face = "bold", size = 12),

axis.title.y.left = element_text(face = "bold", size = 12),

legend.position = "none",

plot.title = element_text(hjust = 0.5, face = "bold", size = 15))

gglist <- c(gglist, list(p))

}

pdf("Figure3_3.pdf", height = 10, width = 8)

cowplot::plot_grid(plotlist = gglist)

dev.off()

# hdWGCNA

options (warn = -1)

library(tidyverse)

library(cowplot)

library(patchwork)

library(WGCNA)

library(hdWGCNA)

theme_set(theme_cowplot())

set.seed(12345)

seu_WGCNA <- SetupForWGCNA(

seu,

group.by = "Group",

gene_select = "fraction", # the gene selection approach

fraction = 0.05, # fraction of cells that a gene needs to be expressed in order to be included

wgcna_name = "hdWGCNA" # the name of the hdWGCNA experiment

)

seu_WGCNA <- MetacellsByGroups(

seurat_obj = seu_WGCNA,

group.by = c("Group"), # specify the columns in seurat_obj@meta.data to group by

k = 20, # nearest-neighbors parameter

max_shared = 10, # maximum number of shared cells between two metacells

ident.group = 'Group', # set the Idents of the metacell seurat object

min_cells = 100

)

#### Removing the following groups that did not meet min_cells: 10 Endothelial_cells, 11 Fibroblasts, 8 Immune_cells, 9 Cardiomyocytes

seu_WGCNA <- NormalizeMetacells(seu_WGCNA)

metacell_seu_WGCNA <- GetMetacellObject(seu_WGCNA)

seu_WGCNA <- NormalizeMetacells(seu_WGCNA)

seu_WGCNA <- ScaleMetacells(seu_WGCNA, features=VariableFeatures(seu_WGCNA))

seu_WGCNA <- RunPCAMetacells(seu_WGCNA, features=VariableFeatures(seu_WGCNA))

seu_WGCNA <- RunHarmonyMetacells(seu_WGCNA, group.by.vars='Group')

seu_WGCNA <- RunUMAPMetacells(seu_WGCNA, reduction='pca', dims=1:20)

p1 <- DimPlotMetacells(seu_WGCNA, group.by='Group') +

ggtitle("Group")

pdf("./hdWGCNA/Figure4_1.pdf", width = 5.5, height = 4.5)

p1

dev.off()

seu_WGCNA <- SetDatExpr(

seu_WGCNA,

group_name = c("MI-3day","Sham-1day"), # the name of the group of interest in the group.by column

group.by = 'Group', # the metadata column containing the cell type info. This same column should have also been used in MetacellsByGroups

assay = 'RNA', # using RNA assay

slot = 'data' # using normalized data

)

# Test different soft powers:

seu_WGCNA <- TestSoftPowers(

seu_WGCNA,

networkType = 'unsigned' # you can also use "unsigned" or "signed hybrid"

)

plot_list <- PlotSoftPowers(seu_WGCNA)

pdf("./hdWGCNA/Figure4_2.pdf", width = 9, height = 9)

wrap_plots(plot_list, ncol=2)

dev.off()

power_table <- GetPowerTable(seu_WGCNA)

seu_WGCNA <- ConstructNetwork(

seu_WGCNA, soft_power = 4,

setDatExpr = FALSE,

networkType = "unsigned",

TOMType = "unsigned",

nThreads = 10,

overwrite_tom = TRUE,

tom_name = 'Tom_Celltype' # name of the topoligical overlap matrix written to disk

)

pdf("./hdWGCNA/Figure4_3.pdf", width = 6.5, height = 5)

PlotDendrogram(seu_WGCNA, main='hdWGCNA Dendrogram')

dev.off()

# TOM <- GetTOM(seu_WGCNA)

seu_WGCNA <- ScaleData(seu_WGCNA, features = rownames(seu_WGCNA))

# compute all MEs in the full single-cell dataset

seu_WGCNA <- ModuleEigengenes(

seu_WGCNA,

group.by.vars = "Group",

exclude_grey = TRUE

)

# harmonized module eigengenes:

hMEs <- GetMEs(seu_WGCNA)

# module eigengenes:

MEs <- GetMEs(seu_WGCNA, harmonized = FALSE)

# compute eigengene-based connectivity (kME):

seu_WGCNA2 <- ModuleConnectivity(

seu_WGCNA,

harmonized = TRUE,

group.by = 'Group', group_name = c("MI-3day","Sham-1day"),

)

# seu_WGCNA2 <- ModuleConnectivity(

# seu_WGCNA,

# harmonized = TRUE,

# group.by = 'Clustes_cellType', group_name = c("5 Macrophages"),

# )

# rename the modules

seu_WGCNA2 <- ResetModuleNames(

seu_WGCNA2,

new_name = "Module"

)

# plot genes ranked by kME for each module

p <- PlotKMEs(seu_WGCNA2, ncol = 3)

pdf("./hdWGCNA/Figure4_4.pdf", width = 6, height = 4)

p

dev.off()

# get the module assignment table:

modules <- GetModules(seu_WGCNA2)

# get hub genes

hub_df <- GetHubGenes(seu_WGCNA2, n_hubs = 20)

saveRDS(seu_WGCNA, file='hdWGCNA_object.rds')

seu_WGCNA2 <- ModuleExprScore(

seu_WGCNA2,

n_genes = 20,

method = 'Seurat'

)

plot_list <- ModuleFeaturePlot(

seu_WGCNA2,

features = 'hMEs', # plot the hMEs

order = FALSE, # order so the points with highest MEs are on top

ucell = FALSE

)

# stitch together with patchwork

pdf("./hdWGCNA/Figure4_5.pdf", width = 9, height = 6)

wrap_plots(plot_list, ncol = 3)

dev.off()

# get hMEs from seurat object

MEs <- GetMEs(seu_WGCNA2, harmonized = TRUE)

mods <- colnames(MEs)

mods <- mods[mods != 'grey']

# add hMEs to Seurat meta-data:

seu_WGCNA2@meta.data <- cbind(seu_WGCNA2@meta.data, MEs)

p <- DotPlot(seu_WGCNA2, features = mods, group.by = 'Group')

p <- p +

coord_flip() +

RotatedAxis() +

scale_color_gradient2(high='red', mid='grey95', low='blue')

pdf("./hdWGCNA/Figure4_6.pdf", width = 4.5, height = 4.5)

p

dev.off()

# Plot hME using Seurat VlnPlot function

Module1_vlnplot <- VlnPlot(

seu_WGCNA2,

features = c('Module1'),

group.by = 'Group',

pt.size = 0 # don't show actual data points

) + geom_boxplot(width=.25, fill='white') +

xlab('') + ylab('hME') + NoLegend() +

scale_fill_manual(values = clusters_color)

Module1_vlnplot

Module2_vlnplot <- VlnPlot(

seu_WGCNA2,

features = c('Module2'),

group.by = 'Group',

pt.size = 0 # don't show actual data points

) + geom_boxplot(width=.25, fill='white') +

xlab('') + ylab('hME') + NoLegend() +

scale_fill_manual(values = clusters_color)

Module2_vlnplot

Module3_vlnplot <- VlnPlot(

seu_WGCNA2,

features = c('Module3'),

group.by = 'Group',

pt.size = 0 # don't show actual data points

) + geom_boxplot(width=.25, fill='white') +

xlab('') + ylab('hME') + NoLegend() +

scale_fill_manual(values = clusters_color)

Module3_vlnplot

Module4_vlnplot <- VlnPlot(

seu_WGCNA2,

features = c('Module4'),

group.by = 'Group',

pt.size = 0 # don't show actual data points

) + geom_boxplot(width=.25, fill='white') +

xlab('') + ylab('hME') + NoLegend() +

scale_fill_manual(values = clusters_color)

Module4_vlnplot

Module5_vlnplot <- VlnPlot(

seu_WGCNA2,

features = c('Module5'),

group.by = 'Group',

pt.size = 0 # don't show actual data points

) + geom_boxplot(width=.25, fill='white') +

xlab('') + ylab('hME') + NoLegend() +

scale_fill_manual(values = clusters_color)

Module5_vlnplot

Module6_vlnplot <- VlnPlot(

seu_WGCNA2,

features = c('Module6'),

group.by = 'Group',

pt.size = 0 # don't show actual data points

) + geom_boxplot(width=.25, fill='white') +

xlab('') + ylab('hME') + NoLegend() +

scale_fill_manual(values = clusters_color) +

stat_compare_means()

Module6_vlnplot

seu_WGCNA2$MI_3day <- ifelse(seu_WGCNA2$Group == "MI-3day",

1, 0)

seu_WGCNA2$Sham_1day <- ifelse(seu_WGCNA2$Group == "Sham-1day",

1, 0)

# list of traits to correlate

cur_traits <- c("MI_3day","Sham_1day")

seu_WGCNA2 <- ModuleTraitCorrelation(

seu_WGCNA2,

traits = cur_traits

)

mt_cor <- GetModuleTraitCorrelation(seu_WGCNA2)

modules_colorBar <- modules[,c("module","color")]

modules_colorBar <- modules_colorBar[!duplicated(modules_colorBar),]

modules_colorBar <- modules_colorBar[-2,]

modules_colorBar_color <- modules_colorBar$color

names(modules_colorBar_color) <- modules_colorBar$module

pdf("./hdWGCNA/Figure4_7.pdf", width = 7, height = 3)

pheatmap(mt_cor$cor$all_cells, breaks = c(-0.3, 0.3),

cluster_cols = F, display_numbers = T, name = "Pearson Correlation",

top_annotation = HeatmapAnnotation(Module = modules_colorBar$module,

col = list(Module = modules_colorBar_color),

show_legend = c(F)

)

)

dev.off()

# PlotModuleTraitCorrelation(

# seu_WGCNA2,

# label = 'Group',

# label_symbol = 'stars',

# text_size = 2,

# text_digits = 2,

# text_color = 'black',

# high_color = 'red',

# mid_color = 'white',

# low_color = 'purple',

# plot_max = 0.5,

# combine = TRUE

# )

Module1_4_6_genes <- modules[which(modules$module %in% c("Module1",

"Module4",

"Module6")), 1:3]

write.table(Module1_4_6_genes$gene_name,

"./hdWGCNA/module_1_4_6_genes.txt",

sep = "\t", row.names = F, col.names = F, quote = F)

string_network <- read.csv("./hdWGCNA/STRING network default node.csv",

header = T)

rownames(string_network) <- string_network$query.term

Module1_4_6_genes$gene_id <- string_network[Module1_4_6_genes$gene_name, "shared.name"]

Module1_4_6_genes <- as.data.frame(na.omit(Module1_4_6_genes))

write.table(Module1_4_6_genes,

"./hdWGCNA/module_1_4_6.txt",

sep = "\t", row.names = F, col.names = T, quote = F)

PPI_100_hub <- read.csv("./hdWGCNA/STRING network_Degree_top100_and_expanded default node.csv", header = T)

PPI_100_hub <- PPI_100_hub$query.term

intersect(PPI_100_hub, rownames(common_genes_diff))

library(VennDiagram)

Tvenn <- venn.diagram(list(MI_3Day_genes = rownames(common_genes_diff),

Module1_Tophub10 = Module1_hub),

filename = NULL,

lwd = 1, lty = 2,

col = c('#20B2AA','#FF4500') ,

fill = c('#20B2AA','#FF4500'),

cat.col = c('black','black'),

reverse = TRUE)

pdf("./hdWGCNA/Figure4_7.pdf", width = 3, height = 3)

grid.draw(Tvenn)

dev.off()

#### CellChat

library(CellChat)

library(patchwork)

options(stringsAsFactors = FALSE)

Idents(seu) <- seu$Group

MI_3day <- subset(seu, idents = "MI-3day")

data.input <- MI_3day@assays$RNA@data

meta <- MI_3day@meta.data

meta$Clustes_cellType <- factor(as.character(meta$Clustes_cellType),

levels = c("1 Fibroblasts","2 Fibroblasts","5 Macrophages","6 Endothelial_cells",

"7 Fibroblasts","8 Immune_cells","9 Cardiomyocytes","10 Endothelial_cells",

"11 Fibroblasts"))

cell.use <- colnames(MI_3day)

CellChatDB <- CellChatDB.mouse

CellChatDB.use <- subsetDB(CellChatDB, search = "Secreted Signaling")

cellchat <- createCellChat(object = data.input, meta = meta, group.by = "Clustes_cellType")

cellchat <- addMeta(cellchat, meta = meta)

cellchat <- setIdent(cellchat, ident.use = "Clustes_cellType")

groupSize <- as.numeric(table(cellchat@idents))

cellchat@DB <- CellChatDB.use

cellchat <- subsetData(cellchat)

future::plan("multiprocess", workers = 14)

cellchat <- identifyOverExpressedGenes(cellchat)

cellchat <- identifyOverExpressedInteractions(cellchat)

cellchat <- projectData(cellchat, PPI.mouse)

cellchat <- computeCommunProb(cellchat, raw.use = FALSE)

cellchat <- filterCommunication(cellchat, min.cells = 10)

df.net <- subsetCommunication(cellchat)

cellchat <- computeCommunProbPathway(cellchat)

cellchat <- aggregateNet(cellchat)

groupSize <- as.numeric(table(cellchat@idents))

pdf("./cellchat/1.all_celltypes_net.pdf", width = 14, height = 8)

par(mfrow = c(1,2), xpd=T)

netVisual_circle(cellchat@net$count, vertex.weight = groupSize, color.use = clusters_color[c(1:2,5:6,7:11)], remove.isolate = F,

weight.scale = T, label.edge= F, title.name = "Number of interactions")

netVisual_circle(cellchat@net$weight, vertex.weight = groupSize, color.use = clusters_color[c(1:2,5:6,7:11)], remove.isolate = F,

weight.scale = T, label.edge= F, title.name = "Interaction weights/strength")

dev.off()

# pdf("./cellchat/1.all_celltypes_net_splited.pdf", width = 15, height = 20)

# mat <- cellchat@net$weight

# par(mfrow = c(3,4), xpd=F)

# for (i in 1:nrow(mat)) {

# mat2 <- matrix(0, nrow = nrow(mat), ncol = ncol(mat), dimnames = dimnames(mat))

# mat2[i, ] <- mat[i, ]

# netVisual_circle(mat2, vertex.weight = groupSize, weight.scale = T, color.use = clusters_color[c(1:2,5:6,7:11)],

# edge.weight.max = max(mat), title.name = rownames(mat)[i], remove.isolate = TRUE)

# }

# dev.off()

cellchat <- netAnalysis_computeCentrality(cellchat, slot.name = "netP")

for (i in unique(df.net$pathway_name)) {

if( !dir.exists(paste0("./cellchat/",i)) ) {

dir.create(paste0("./cellchat/",i))

}

pathways.show <- i

vertex.receiver <- seq(1,4)

pdf(paste0("./cellchat/",i,"/2.",i,"_circle.pdf"), width = 8, height = 8)

par(mfrow=c(1,1))

p <- netVisual_aggregate(cellchat, signaling = pathways.show, layout = "circle", color.use = clusters_color[c(1:2,5:6,7:11)])

print(p)

dev.off()

pdf(paste0("./cellchat/",i,"/2.",i,"_heatmap.pdf"), width = 8, height = 8)

par(mfrow=c(1,1))

p <- netVisual_heatmap(cellchat, signaling = pathways.show, color.heatmap = "Reds", color.use = clusters_color[c(1:2,5:6,7:11)])

print(p)

dev.off()

height <- length(unique(df.net[which(df.net$pathway_name == i),"interaction_name_2"]))

pdf(paste0("./cellchat/",i,"/2.",i,"_LR_contribution.pdf"), width = 5, height = 1.5*height)

p <- netAnalysis_contribution(cellchat, signaling = pathways.show)

print(p)

dev.off()

pairLR <- extractEnrichedLR(cellchat, signaling = pathways.show, geneLR.return = T)

LR.show <- pairLR$pairLR[1,]

vertex.receiver <- seq(1,4)

pdf(paste0("./cellchat/",i,"/2.",i,"_enriched_LR.pdf"), width = 6, height = 6)

p <- netVisual_individual(cellchat, signaling = pathways.show, pairLR.use = LR.show,

vertex.receiver = vertex.receiver, color.use = clusters_color[c(1:2,5:6,7:11)])

print(p)

dev.off()

pathways.show.all <- cellchat@netP$pathways

levels(cellchat@idents)

vertex.receiver <- seq(1,4)

pdf(paste0("./cellchat/",i,"/2.",i,"_path_T_to_others.pdf"), width = 4, height = 2+0.5*height)

p <- netVisual_bubble(cellchat, sources.use = c(1:2,4:9), targets.use = 3, signaling = i, remove.isolate = F)

print(p)

dev.off()

pathway_genes <- unique(c(df.net[which(df.net$pathway_name == i),"ligand"],

df.net[which(df.net$pathway_name == i),"receptor"]))

pdf(paste0("./cellchat/",i,"/2.",i,"_gene_expression.pdf"), width = 6.5, height = 1+1*length(pathway_genes))

p <- plotGeneExpression(cellchat, signaling = i, color.use = clusters_color[c(1:2,5:6,7:11)])

print(p)

dev.off()

pdf(paste0("./cellchat/",i,"/2.",i,"_signalingRole_network.pdf"), width = 8, height = 4)

p <- netAnalysis_signalingRole_network(cellchat, signaling = pathways.show, width = 8, height = 2.5, font.size = 10, color.use = clusters_color[c(1:2,5:6,7:11)])

print(p)

dev.off()

}

# g <- HubGeneNetworkPlot(seu_WGCNA,

# n_hubs = 5, n_other = 20,

# edge_prop = 0.9,

# mods = c("Module1", "Module3", "Module5"),

# return_graph = FALSE)

# g

# seu_WGCNA <- RunModuleUMAP(

# seu_WGCNA,

# n_hubs = 10, # number of hub genes to include for the UMAP embedding

# n_neighbors = 15, # neighbors parameter for UMAP

# min_dist = 0.1 # min distance between points in UMAP space

# )

#

# umap_df <- GetModuleUMAP(seu_WGCNA)

#

# ggplot(umap_df, aes(x = UMAP1, y = UMAP2)) +

# geom_point(

# color = umap_df$color, # color each point by WGCNA module

# size = umap_df$kME*2 # size of each point based on intramodular connectivity

# )

#

# ModuleUMAPPlot(

# seu_WGCNA,

# edge.alpha = 0.25,

# sample_edges = TRUE,

# edge_prop = 0.1, # proportion of edges to sample (20% here)

# label_hubs = 5, # how many hub genes to plot per module?

# keep_grey_edges = FALSE

#

# )

###### 不用7day数据

# setwd("E:\\xianyu\\chenliyuan\\bulk_7d")

# library(GenomicFeatures)

# txdb <- makeTxDbFromGFF("gencode.vM25.basic.annotation.gtf",format="gtf")

# exons_gene <- exonsBy(txdb, by = "gene")

# exons_gene_lens <- lapply(exons_gene,function(x){sum(width(reduce(x)))})

# gene_length <- sapply(exons_gene_lens,function(x){x})

# id_length <- as.data.frame(gene_length)

#

# gene_id <- read.csv("ensembl_Gene_length.txt",

# header = T, sep = "\t")

# gene_id <- gene_id[,c("Gene.stable.ID", "Gene.name")]

# gene_id <- gene_id[!duplicated(gene_id$Gene.name),]

# id_length$id <- unlist(lapply(rownames(id_length), function(x){

# unlist(strsplit(x, ".", fixed = T))[1]

# }))

#

# id_length$symbol <- gene_id[match(id_length$id, gene_id$Gene.stable.ID),"Gene.name"]

# id_length <- as.data.frame(na.omit(id_length))

#

# rownames(id_length) <- id_length$symbol

#

# GSE181872 <- read.csv("GSE181872_Raw_gene_counts_matrix.txt",

# sep = "\t", header = T, row.names = 1, check.names = F)

# GSE181872 <- aggregate.data.frame(GSE181872[,2:ncol(GSE181872)],

# by = list(GSE181872$Symbol),

# FUN = max)

# rownames(GSE181872) <- GSE181872$Group.1

# GSE181872 <- GSE181872[,-1]

# GSE181872 <- as.data.frame(cbind(Length = id_length[rownames(GSE181872),1],

# GSE181872))

# GSE181872 <- as.data.frame(na.omit(GSE181872))

# countToFpkm <- function(counts, effLen) {

# N <- sum(counts)

# exp( log(counts) + log(1e9) - log(effLen) - log(N) )

# }

# for (i in 2:ncol(GSE181872)) {

# GSE181872[,i] <- countToFpkm(GSE181872[,i], GSE181872$Length)

# }

# GSE181872 <- GSE181872[,-1]

# GSE181872 <- log2(GSE181872+1)

# GSE181872 <- GSE181872[,c("MI (Day 7)_1","MI (Day 7)_2","MI (Day 7)_3","MI (Day 7)_4")]

# colnames(GSE181872) <- c("GSE181872-MI_7Day1", "GSE181872-MI_7Day2",

# "GSE181872-MI_7Day3", "GSE181872-MI_7Day4")

#

# GSE114695 <- read.csv("GSE114695_Gene_FC_P-val_FPKM.txt",

# sep = "\t", header = T, check.names = F)

# GSE114695 <- GSE114695[,c(3,53:70)]

# GSE114695 <- aggregate.data.frame(GSE114695[,2:ncol(GSE114695)],

# by = list(GSE114695$Gene_Symbol),

# FUN = max)

# GSE114695 <- GSE114695[-1,]

# rownames(GSE114695) <- GSE114695$Group.1

# GSE114695 <- GSE114695[,-1]

# GSE114695 <- log2(GSE114695 + 1)

# GSE114695 <- GSE114695[,c("MI_1w-10_FPKM","MI_1w-2_FPKM","MI_1w-6_FPKM")]

# colnames(GSE114695) <- c("GSE114695-MI_7Day1", "GSE114695-MI_7Day2", "GSE114695-MI_7Day3")

#

# keep_num <- c()

# for (i in 1:nrow(GSE181872)) {

# temp <- GSE181872[i,] == 0

# sample_0 <- sum(temp[1,])

# if (sample_0 <= 1) {

# keep_num <- c(keep_num, i)

# }

# }

# GSE181872 <- GSE181872[keep_num,]

#

# keep_num <- c()

# for (i in 1:nrow(GSE114695)) {

# temp <- GSE114695[i,] == 0

# sample_0 <- sum(temp[1,])

# if (sample_0 <= 1) {

# keep_num <- c(keep_num, i)

# }

# }

# GSE114695 <- GSE114695[keep_num,]

#

# GSE206281_7day <- GSE206281[,c("GSE206281-MI_3Day1", "GSE206281-MI_3Day2", "GSE206281-MI_3Day3")]

# GSE153494_7day <- GSE153494[,c("GSE153494-MI_3Day1", "GSE153494-MI_3Day2", "GSE153494-MI_3Day3")]

#

# keep_num <- c()

# for (i in 1:nrow(GSE206281_7day)) {

# temp <- GSE206281_7day[i,] == 0

# sample_0 <- sum(temp[1,])

# if (sample_0 <= 1) {

# keep_num <- c(keep_num, i)

# }

# }

# GSE206281_7day <- GSE206281_7day[keep_num,]

#

# keep_num <- c()

# for (i in 1:nrow(GSE153494_7day)) {

# temp <- GSE153494_7day[i,] == 0

# sample_0 <- sum(temp[1,])

# if (sample_0 <= 1) {

# keep_num <- c(keep_num, i)

# }

# }

# GSE153494_7day <- GSE153494_7day[keep_num,]

#

#

# common_genes <- intersect(rownames(GSE181872), rownames(GSE114695))

# common_genes <- intersect(common_genes, rownames(GSE206281_7day))

# common_genes <- intersect(common_genes, rownames(GSE153494_7day))

#

# mergeData_2 <- as.data.frame(cbind(

# GSE181872[common_genes,],

# GSE114695[common_genes,],

# GSE206281_7day[common_genes,],

# GSE153494_7day[common_genes,]

# ))

#

# group <- as.factor(c(rep("GSE181872-MI_7Day",4),

# rep("GSE114695-MI_7Day",3),

# rep("GSE206281-MI_3Day",3),

# rep("GSE153494-MI_3Day",3)))

#

# pca_before_2 <- prcomp(as.data.frame(t(mergeData_2)), scale = TRUE)

#

# P1_2 <- fviz_pca_ind(pca_before_2, label = "none",

# habillage = group, addEllipses = FALSE,

# palette = c("#FF6666",

# "#3366FF",

# "#FF9933",

# "#99CCFF")) +

# theme_classic() +

# theme(plot.title = element_text(hjust = 0.5, face = "bold"),

# legend.position = "none") +

# ggtitle(label = "Before removing batch effect") +

# ggforce::geom_mark_ellipse(aes(color = group))

#

# batch <- factor(c(rep("GSE181872", 4),

# rep("GSE114695", 3),

# rep("GSE206281", 3),

# rep("GSE153494", 3)))

#

# # mod <- as.factor(c(rep("Day7", 7), rep("Day3", 6)))

# # mod <- model.matrix(~mod)

#

# combat_data_2 <- ComBat(dat = mergeData_2, batch = batch)

#

# combat_data_2[which(combat_data_2 < 0)] <- 0

# combat_data_2 <- as.data.frame(combat_data_2)

# pca_after_combat_2 <- prcomp(as.data.frame(t(combat_data_2)), scale = TRUE)

#

# P2_2 <- fviz_pca_ind(pca_after_combat_2, label = "none",

# habillage = group, addEllipses = FALSE,

# palette = c("#FF6666",

# "#3366FF",

# "#FF9933",

# "#99CCFF")) +

# theme_classic() +

# theme(plot.title = element_text(hjust = 0.5, face = "bold")) +

# ggtitle(label = "After removing batch effect") +

# ggforce::geom_mark_ellipse(aes(color = group))

# P1_2 + P2_2

#

# diff_3_7 <- c()

# for (i in intersect(rownames(common_genes_diff), rownames(mergeData_2))) {

# fc <- sum(t(mergeData_2)[c(8:13),i]) / sum(t(mergeData_2)[c(1:7),i])

# p <- wilcox.test(t(mergeData_2)[c(1:7),i], t(mergeData_2)[c(8:13),i])

# p <- p$p.value

# diff_3_7 <- as.data.frame(rbind(diff_3_7,

# data.frame(gene = i,

# fc = fc,

# p = p)))

# }

#

library(biomaRt)

IRGs <- read.csv("./2-18/GeneCards-SearchResults.csv", header = T)

human <- useMart("ensembl", dataset = "hsapiens_gene_ensembl", host = "https://dec2021.archive.ensembl.org/")

mouse <- useMart("ensembl", dataset = "mmusculus_gene_ensembl", host = "https://dec2021.archive.ensembl.org/")

IRGs_mouse <- getLDS(attributes = c("mgi_symbol"), filters = "mgi_symbol",

values = IRGs$Gene.Symbol,

mart = mouse,

attributesL = c("hgnc_symbol"),

martL = human, uniqueRows = T)

library(VennDiagram)

Tvenn <- venn.diagram(list(IRGs = IRGs_mouse$MGI.symbol,

"MI-3d Genes" = common_genes),

filename=NULL,

lwd=1,lty=1,

col=c('black','black'),

fill=c("#20B2AA",'#FF7F50'),

cat.col=c('black','black'),

reverse=TRUE)

pdf("./2-18/3.19genes_venn.pdf", width = 4.5, height = 4.5)

grid.draw(Tvenn)

dev.off()

common_genes

intersect(common_genes, IRGs_mouse$MGI.symbol)

Gene_drug <- read.table("./2-18/dgidb_export_2023-02-19.tsv",

sep = "\t", header = T)

Gene_drug <- data.frame(term = c(unique(Gene_drug$gene),

unique(Gene_drug$drug)),

type = c(rep("Gene",length(unique(Gene_drug$gene))),

rep("Drug",length(unique(Gene_drug$drug)))))

write.table(Gene_drug, "./2-18/Gene_drug.txt", sep = "\t", quote = F, row.names = F)

## ImmucellAI

library(ggcor)

library(vegan)

library(dplyr)

library(ggplot2)

library(ggpubr)

hubGenes <- c("Grn","Igf1","Il18","Itgb2","Ncf2","Ncf4","Spp1" )

hub_data <- t(combat_data_immu)[,hubGenes]

hub_data <- hub_data[-1,]

hub_data_2 <- apply(hub_data, 2, as.numeric)

rownames(hub_data_2) <- rownames(hub_data)

set.seed(20211130)

mantel <- mantel_test(as.data.frame(hub_data_2)+0.001, as.data.frame(t(abundance_cell)[rownames(hub_data_2),]),

spec.select = list(Grn = 1,

Igf1 = 2,

Il18 = 3,

Itgb2 = 4,

Ncf2 = 5,

Ncf4 = 6,

Spp1 = 7)) %>%

mutate(r = cut(r, breaks = c(-Inf, 0, Inf),

labels = c("r <= 0", "r > 0")),

p.value = cut(p.value, breaks = c(0, 0.01, 0.05, 1),

labels = c("< 0.01", "< 0.05", ">= 0.05"),

right = FALSE, include.lowest = TRUE))

corr <- fortify_cor(t(abundance_cell), type = "upper", show.diag = TRUE,

cor.test = TRUE, cluster.type = "all")

#pdf("E:/xianyu/chenliyuan/2-18/7.immu_cor.pdf", height = 8, width = 12)

pdf("E:/xianyu/chenliyuan/ImmuCellAI/immu_cor.pdf", height = 8, width = 12)

quickcor(t(abundance_cell), type = "upper", cluster = TRUE, show.diag = T, axis.x.position = "top") + geom_square() +

scale_fill_gradient2(low = "#0000CC", mid = "white", high = "#CC0033") +

add_link(mantel, mapping = aes(colour = p.value, size = r),

diag.label = F) +

scale_size_manual(values = c(0.5, 1, 2)) +

scale_color_manual(values = c("#FF3300", "#FF6699", "#CCCCCC"))

dev.off()

pdf("../2-18/8.hubGenes_featureplots.pdf", height = 4, width = 16)

p1 <- FeaturePlot(seu, features = hubGenes, order = T, ncol = 4)

print(p1)

dev.off()

pdf("./验证/Serpina3n_FeaturePlot.pdf")

p1 <- FeaturePlot(seu, features = "Serpina3n", order = T)

print(p1)

dev.off()

pdf("./验证/Serpina3n_vlnplotplots.pdf", height = 4.5, width = 6)

p2 <- VlnPlot(seu, features = "Serpina3n", flip = F, group.by = "Clustes_cellType") +

NoLegend()

print(p2)

dev.off()

p2 <- VlnPlot(seu, features = hubGenes, flip = T, stack = T, group.by = "Clustes_cellType") +

NoLegend()

pdf("../2-18/8.hubGenes_vlnplotplots.pdf", height = 4, width = 8)

print(p2)

dev.off()

library(corrplot)

library(RColorBrewer)

color_1 <- rev(brewer.pal(10,"RdYlGn"))

pdf("E:/xianyu/chenliyuan/免疫补充/单细胞figures/11.hubGenes_cor.pdf", width = 3.5, height = 3)

corrplot.mixed(cor(hub_data_2), lower.col = color_1, upper.col = color_1, order = 'AOE')

dev.off()

library(limma)

library(ggplot2)

library(forcats)

library(ggstance)

library(clusterProfiler)

library(msigdbr)

library(enrichplot)

library(ggstatsplot)

set.seed(12345)

C2 <- msigdbr(species = "Mus musculus", category = "C2")

table(C2$gs_subcat)

C2 <- subset(C2, gs_subcat=="CP:KEGG")

for (gene in hubGenes) {

print(gene)

temp <- hub_data_2[7:12,gene]

group <- ifelse(temp > median(temp),

"High", "Low")

group <- factor(group, levels = c("High", "Low"))

data <- combat_data_immu[-1,-1][,names(group)]

data2 <- apply(data, 2, as.numeric)

rownames(data2) <- rownames(data)

data2 <- t(data2)

fc <- apply(data2, 2, function(x){

(mean(x[which(group == "High")])+0.001) / (mean(x[which(group == "Low")])+0.001)

})

fc <- log2(fc)

diff_list <- sort(fc, decreasing = T)

KEGG_gsea <- GSEA(diff_list, TERM2GENE = C2[,c(15,4)], verbose = T, eps = 0, pvalueCutoff = 1)

KEGG_gsea_result <- as.data.frame(KEGG_gsea@result)

KEGG_gsea_result <- KEGG_gsea_result[which(KEGG_gsea_result$pvalue < 0.05),]

KEGG_pathway <- C2[,c(13,15)]

KEGG_pathway <- KEGG_pathway[!duplicated(KEGG_pathway),]

KEGG_gsea_result$ID <- KEGG_pathway$gs_exact_source[match(KEGG_gsea_result$ID, KEGG_pathway$gs_description)]

rownames(KEGG_gsea_result) <- KEGG_gsea_result$ID

KEGG_gsea@result <- KEGG_gsea_result

KEGG_gsea_result <- KEGG_gsea_result[order(KEGG_gsea_result$NES, decreasing = T),]

path <- "E:/xianyu/chenliyuan/免疫补充/单细胞figures/GSEA/"

if (!dir.exists(paste0(path, gene))) {

dir.create(paste0(path, gene))

}

saveRDS(KEGG_gsea, paste0(path, gene, "/", gene, ".rds"))

write.table(KEGG_gsea_result, paste0(path, gene, "/KEGG_gsea_result.txt"),

sep = "\t", row.names = F, col.names = T, quote = F)

rownames(KEGG_gsea@result) <- KEGG_gsea@result$Description

KEGG_gsea@result$ID <- KEGG_gsea@result$Description

for (i in KEGG_gsea@result$ID) {

pdf(paste0(path, gene, "/", gsub(pattern = "/", replacement = "", i, fixed = T),".pdf"), height = 6, width = 8)

gsea_plot <- gseaplot2(KEGG_gsea,

i, #绘制i通路

color="red", #线条颜色

base_size = 20, #基础字体的大小

subplots = 1:3, #展示上2部分

pvalue_table = T,

title = i) # 显示p值

gsea_plot <- gsea_plot + theme(plot.title = element_text(vjust = 0.5))

print(gsea_plot)

dev.off()

}

}

GSEA_data <- readRDS("E:\\xianyu\\chenliyuan\\2-18\\GSEA\\Mmp9\\Mmp9.rds")

rownames(GSEA_data@result) <- GSEA_data@result$Description

GSEA_data@result$ID <- GSEA_data@result$Description

gsea_plot <- gseaplot2(GSEA_data,

c("NOD-like receptor signaling pathway",

"Cytokine-cytokine receptor interaction",

"Chemokine signaling pathway",

"Leukocyte transendothelial migration",

"Toll-like receptor signaling pathway",

"Cell adhesion molecules (CAMs)"), #绘制i通路

base_size = 20, #基础字体的大小

subplots = 1:3, #展示上2部分

pvalue_table = T,

title = "Mmp9")

gsea_plot <- gsea_plot + theme(plot.title = element_text(vjust = 0.5))

pdf("E:\\xianyu\\chenliyuan\\2-18\\GSEA\\Mmp9_GSEA.pdf", width = 5.5, height = 5)

print(gsea_plot)

dev.off()

Part 2 codes for hdWGCNA.R

setwd("E:/xianyu/chenliyuan/WGCNA")

library(WGCNA)

library(reshape2)

library(stringr)

options(stringsAsFactors = FALSE)

enableWGCNAThreads() # 打开多线程

Matrix <- as.data.frame(t(combat_data)) # 行是sample，列是gene

gsg <- goodSamplesGenes(Matrix, verbose = 3) # 过滤低质量的基因和样本

if (!gsg$allOK)

{

# 打印被删除的基因和样本名称

if (sum(!gsg$goodGenes)>0)

printFlush(paste("Removing genes:", paste(names(datExpr0)[!gsg$goodGenes], collapse = ", ")));

if (sum(!gsg$goodSamples)>0)

printFlush(paste("Removing samples:", paste(rownames(datExpr0)[!gsg$goodSamples], collapse = ", ")));

# 从数据中移除有害基因和样本

Matrix <- Matrix[gsg$goodSamples, gsg$goodGenes]

}

Matrix <- Matrix[gsg$goodSamples, gsg$goodGenes]

# 筛选sd排名前几的基因

allgene_sd <- apply(Matrix, 2, sd)

allgene_sd <- sort(allgene_sd, decreasing = T)

# Matrix <- Matrix[,names(which(allgene_sd>=quantile(allgene_sd)[4]))] # 3533个基因

Matrix <- Matrix[,names(allgene_sd)[1:5000]] # 2557个基因

# 输入临床数据

traitData <- data.frame(Group = c(rep("Sham", 3), rep("MI_10m", 3), rep("MI_1h", 3), rep("MI_6h", 3), rep("MI_1day", 3), rep("MI_3day", 3),

rep("Sham", 3), rep("MI_10m", 3), rep("MI_1h", 3), rep("MI_6h", 3), rep("MI_1day", 3), rep("MI_3day", 3)),

Sample = rownames(Matrix),

row.names = rownames(Matrix))

traitData$Group <- factor(traitData$Group, levels = c("Sham", "MI_10m", "MI_1h", "MI_6h", "MI_1day", "MI_3day"))

traitData <- traitData[order(traitData$Group),]

Matrix <- as.data.frame(Matrix[rownames(traitData),])

# 将特征转换为颜色表示

traitColors <- data.frame(

Sham = c(rep("#4169E1",6), rep("#FFFFFF",30)),

MI_10m = c(rep("#FFFFFF",6), rep("#545AE3",6), rep("#FFFFFF",24)),

MI_1h = c(rep("#FFFFFF",12), rep("#674BE7",6), rep("#FFFFFF",18)),

MI_6h = c(rep("#FFFFFF",18), rep("#7A3DEA",6), rep("#FFFFFF",12)),

MI_1day = c(rep("#FFFFFF",24), rep("#8D2EED",6), rep("#FFFFFF",6)),

MI_3day = c(rep("#FFFFFF",30), rep("#A020F0",6))

)

sampleTree <- hclust(dist(Matrix), method = "average")

# 绘制样本聚类图和对应的颜色

pdf("SI_4_samples_hclust.pdf")

plotDendroAndColors(sampleTree, traitColors,

groupLabels = c("Sham","MI_10m","MI_1h","MI_6h","MI_1day","MI_3day"),

main = "Sample dendrogram and trait heatmap",

autoColorHeight = FALSE,

colorHeight = 0.2)

dev.off()

# 去除离群样本

# sizeGrWindow(12,9)

# pdf(file = "Plots/1.sampleClustering.pdf", width = 12, height = 9);

# par(cex = 0.6)

# par(mar = c(0,4,2,0))

# plot(sampleTree, main = "Sample clustering to detect outliers", sub="", xlab="", cex.lab = 1.5,

# cex.axis = 1.5, cex.main = 2)

# 画一条线来表示切割

# abline(h = 150, col = "red")

# Determine cluster under the line

# clust <- cutreeStatic(sampleTree, cutHeight = 190, minSize = 10)

# table(clust)

# clust 1 包含了我们想要的sample

# keepSamples <- c(clust==1)

# datExpr <- Matrix[keepSamples, ]

datExpr <- Matrix

nGenes <- ncol(datExpr)

nSamples <- nrow(datExpr)

#构建临床性状

datTraits <- matrix(as.numeric(traitColors != "#FFFFFF"), byrow = F, ncol = 6)

datTraits <- as.data.frame(datTraits)

colnames(datTraits) <- colnames(traitColors)

rownames(datTraits) <- rownames(traitData)

collectGarbage()

# 选择一组软阈值

powers <- 1:100

# 网络拓扑分析

sft <- pickSoftThreshold(datExpr, powerVector = powers, verbose = 5)

# Plot the results:

pdf("Figure2_4.确定软阈值.pdf", width = 8, height = 6.5)

par(mfrow = c(1,2))

cex1 <- 0.9

# Scale-free topology fit index as a function of the soft-thresholding power

softPower <- sft$powerEstimate

adjacency <- adjacency(datExpr, power = softPower)

plot(sft$fitIndices[,1], -sign(sft$fitIndices[,3])*sft$fitIndices[,2],

xlab="Soft Threshold (power)",ylab="Scale Free Topology Model Fit,signed R^2",type="n",

main = paste("Scale independence"));

text(sft$fitIndices[,1], -sign(sft$fitIndices[,3])*sft$fitIndices[,2],

labels=powers,cex=cex1,col="red");

# this line corresponds to using an R^2 cut-off of h

h <- sft[["fitIndices"]][["SFT.R.sq"]][softPower]

abline(h=0.85,col="red")

# Mean connectivity as a function of the soft-thresholding power

plot(sft$fitIndices[,1], sft$fitIndices[,5],

xlab="Soft Threshold (power)",ylab="Mean Connectivity", type="n",

main = paste("Mean connectivity"))

text(sft$fitIndices[,1], sft$fitIndices[,5], labels=powers, cex=cex1,col="red")

dev.off()

TOM <- TOMsimilarity(adjacency, TOMType = "unsigned")

colnames(TOM) <- colnames(adjacency)

rownames(TOM) <- rownames(adjacency)

dissTOM <- 1-TOM

# 基因聚类

geneTree <- hclust(as.dist(dissTOM), method = "average");

# 绘制聚类树

sizeGrWindow(12,9)

plot(geneTree, xlab="", sub="", main = "Gene clustering on TOM-based dissimilarity",

labels = FALSE, hang = 0.04)

minModuleSize <- 50

# 使用动态树切割识别模块

dynamicMods <- cutreeDynamic(dendro = geneTree, distM = dissTOM,

deepSplit = 2, pamRespectsDendro = FALSE,

minClusterSize = minModuleSize);

table(dynamicMods)

# 将数字标签转换成颜色

dynamicColors <- labels2colors(dynamicMods)

table(dynamicColors)

# 给聚类树加上颜色

sizeGrWindow(8,6)

plotDendroAndColors(geneTree, dynamicColors, "Dynamic Tree Cut",

dendroLabels = FALSE, hang = 0.03,

addGuide = TRUE, guideHang = 0.05,

main = "Gene dendrogram and module colors")

# 计算 eigengenes

MEList <- moduleEigengenes(datExpr, colors = dynamicColors)

MEs <- MEList$eigengenes

# 计算模块 eigengenes 之间的距离

MEDiss <- 1 - cor(MEs)

# Cluster module eigengenes

METree <- hclust(as.dist(MEDiss), method = "average")

# Plot the result

sizeGrWindow(7, 6)

plot(METree, main = "Clustering of module eigengenes",

xlab = "", sub = "")

MEDissThres <- 0.3 # 模块合并阈值

# Plot the cut line into the dendrogram

abline(h=MEDissThres, col = "red")

# Call an automatic merging function

merge <- mergeCloseModules(datExpr, dynamicColors, cutHeight = MEDissThres, verbose = 3)

# The merged module colors

mergedColors <- merge$colors

# Eigengenes of the new merged modules:

mergedMEs <- merge$newMEs

sizeGrWindow(12, 9)

pdf("2.基因聚类模块.pdf", wi = 9, he = 6)

plotDendroAndColors(geneTree, cbind(dynamicColors, mergedColors),

c("Dynamic Tree Cut", "Merged dynamic"),

dendroLabels = FALSE, hang = 0.03,

addGuide = TRUE, guideHang = 0.05)

dev.off()

# Rename to moduleColors

moduleColors <- mergedColors

# Construct numerical labels corresponding to the colors

colorOrder <- c("grey", standardColors(50))

moduleLabels <- match(moduleColors, colorOrder)-1

MEs <- mergedMEs

# Recalculate MEs with color labels

MEs0 <- moduleEigengenes(datExpr, moduleColors)$eigengenes

MEs <- orderMEs(MEs0)

datTraits <- datTraits[rownames(MEs),]

moduleTraitCor <- cor(MEs, datTraits, use = "p")

moduleTraitPvalue <- corPvalueStudent(moduleTraitCor, nSamples)

sizeGrWindow(10,6)

# Will display correlations and their p-values

textMatrix <- paste(signif(moduleTraitCor, 2), "\n(",

signif(moduleTraitPvalue, 1), ")", sep = "")

dim(textMatrix) <- dim(moduleTraitCor)

par(mar = c(6, 8.5, 3, 3))

# Display the correlation values within a heatmap plot

pdf("3.临床性状和模块的相关性.pdf")

labeledHeatmap(Matrix = moduleTraitCor,

xLabels = names(datTraits),

yLabels = names(MEs),

ySymbols = names(MEs),

colorLabels = FALSE,

colors = greenWhiteRed(50),

textMatrix = textMatrix,

setStdMargins = FALSE,

cex.text = 0.5,

zlim = c(-1,1),

main = paste("Module-trait relationships"))

dev.off()

#计算MM和GS值

modNames <- substring(names(MEs), 3)

geneModuleMembership <- as.data.frame(cor(datExpr, MEs, use = "p"))

MMPvalue <- as.data.frame(corPvalueStudent(as.matrix(geneModuleMembership), nSamples))

names(geneModuleMembership) <- paste("MM", modNames, sep="")

names(MMPvalue) <- paste("p.MM", modNames, sep="")

traitNames <- names(datTraits)

geneTraitSignificance <- as.data.frame(cor(datExpr, datTraits, use = "p"))

GSPvalue <- as.data.frame(corPvalueStudent(as.matrix(geneTraitSignificance), nSamples))

names(geneTraitSignificance) <- paste("GS.", traitNames, sep="")

names(GSPvalue) <- paste("p.GS.", traitNames, sep="")

#批量输出性状和模块基因散点图

for (trait in traitNames){

traitColumn <- match(trait,traitNames)

for (module in modNames){

column <- match(module, modNames)

moduleGenes <- moduleColors==module

if (nrow(geneModuleMembership[moduleGenes,]) > 1){

outPdf=paste(trait, "_", module,".pdf",sep="")

pdf(file=outPdf,width=7,height=7)

par(mfrow = c(1,1))

verboseScatterplot(abs(geneModuleMembership[moduleGenes, column]),

abs(geneTraitSignificance[moduleGenes, traitColumn]),

xlab = paste("Module Membership in", module, "module"),

ylab = paste("Gene significance for ",trait, "(abs)"),

main = paste("Module membership vs. gene significance\n"),

cex.main = 1.2, cex.lab = 1.2, cex.axis = 1.2, col = module, pch = 19, cex = 0.8)

abline(v=0.7,h=0.5,col="red")

dev.off()

}

}

}

#输出GS-MM数据

probes <- colnames(datExpr)

geneInfo0 <- data.frame(probes= probes,

moduleColor = moduleColors)

for (Tra in 1:ncol(geneTraitSignificance)) {

oldNames <- names(geneInfo0)

geneInfo0 <- data.frame(geneInfo0, geneTraitSignificance[,Tra],

GSPvalue[, Tra])

names(geneInfo0) <- c(oldNames,names(geneTraitSignificance)[Tra],

names(GSPvalue)[Tra])

}

for (mod in 1:ncol(geneModuleMembership)) {

oldNames <- names(geneInfo0)

geneInfo0 <- data.frame(geneInfo0, geneModuleMembership[,mod],

MMPvalue[, mod])

names(geneInfo0) <- c(oldNames,names(geneModuleMembership)[mod],

names(MMPvalue)[mod])

}

geneOrder <- order(geneInfo0$moduleColor)

geneInfo <- geneInfo0[geneOrder, ]

write.table(geneInfo, file = "GS_MM.xls",sep="\t",row.names=F)

#输出每个模块的基因

for (mod in 1:nrow(table(moduleColors))) {

modules <- names(table(moduleColors))[mod]

probes <- colnames(datExpr)

inModule <- (moduleColors == modules)

modGenes <- probes[inModule]

write.table(modGenes, file =paste0("LIHC_",modules,".txt"),sep="\t",row.names=F,col.names=F,quote=F)

}

#选择royalblue、cyan、brown三种模块中的显著的关键基因

royalblue <- rownames(geneModuleMembership)[moduleColors=="royalblue"]

cyan <- rownames(geneModuleMembership)[moduleColors=="cyan"]

brown <- rownames(geneModuleMembership)[moduleColors=="brown"]

royalblue_2 <- rownames(geneModuleMembership[royalblue,])[(geneModuleMembership[royalblue,]$MMroyalblue > 0.7)]

royalblue_hub <- rownames(geneTraitSignificance[royalblue_2,])[(abs(geneTraitSignificance[royalblue_2,]$GS.Case) > 0.5)]

cyan_2 <- rownames(geneModuleMembership[cyan,])[(geneModuleMembership[cyan,]$MMcyan > 0.7)]

cyan_hub <- rownames(geneTraitSignificance[cyan_2,])[(abs(geneTraitSignificance[cyan_2,]$GS.Case) > 0.5)]

brown_2 <- rownames(geneModuleMembership[brown,])[(geneModuleMembership[brown,]$MMbrown > 0.7)]

brown_hub <- rownames(geneTraitSignificance[brown_2,])[(abs(geneTraitSignificance[brown_2,]$GS.Case) > 0.5)]

### 可视化

# 模块相似性

plotEigengeneNetworks(MEs, "Eigengene adjacency heatmap",

marDendro = c(3,3,2,4),

marHeatmap = c(3,4,2,2), plotDendrograms = T,

xLabelsAngle = 90)

# 选择一部分模块基因做热图

select <- which(moduleColors %in% c("green","brown","red"))

selectTOM <- dissTOM[select, select]

selectTree <- hclust(as.dist(selectTOM), method = "average")

selectColors <- moduleColors[select]

# Open a graphical window

sizeGrWindow(9,9)

plotDiss <- selectTOM^softPower

diag(plotDiss) <- NA

library(RColorBrewer)

library(momr)

color <- rev(colorRampPalette(c("#FFFF99","#FFCC33", "#FF3333","#CC3300"))(100))

plotCol(color)

TOMplot(plotDiss, selectTree, selectColors,

main = "Network heatmap plot, selected genes",

col = color)

Part three code for ROC.R

###########

library(pROC)

library(ROCR)

library(tidyverse)

library(cowplot)

rm(list = ls())

setwd("/public/home/wuyf/project/singlecell/RX2025-12010/analysis/GSE60993")

GPL6884 <- read.csv("GPL6884-11607.txt", sep = "\t", header = T, row.names = 1)

GPL6884 <- GPL6884[-which(is.na(GPL6884$Entrez_Gene_ID)),]

GSE60993 <- read.csv("GSE60993_non-normalized.txt", sep = "\t", row.names = 1)

GSE60993 <- GSE60993[,grep(pattern = "AVG_Signal", colnames(GSE60993))]

GSE60993_1 <- select(GSE60993,starts_with("Normal"))

GSE60993_2 <- select(GSE60993,starts_with("STEMI"))

GSE60993 <- cbind(GSE60993_1,GSE60993_2)

GSE60993_meta <- data.frame(symbol = colnames(GSE60993),

group = colnames(GSE60993))

GSE60993_meta$group <- unlist(lapply(GSE60993_meta$group, function(x){

unlist(strsplit(x, "_"))[1]

}))

table(GSE60993_meta$group)

GSE60993_meta$group[which(GSE60993_meta$group %in% c("STEMI"))] <- "IM"

rownames(GSE60993_meta) <- GSE60993_meta$symbol

GSE60993 <- data.frame(symbol = GPL6884[rownames(GSE60993), "ILMN_Gene"],

GSE60993)

GSE60993 <- aggregate.data.frame(GSE60993[,2:ncol(GSE60993)],

by = list(GSE60993$symbol),

FUN = mean)

rownames(GSE60993) <- GSE60993$Group.1

GSE60993 <- GSE60993[,-1]

keygenes <- c("Grn", "Igf1", "Il18", "Itgb2", "Ncf2", "Ncf4", "Spp1")

keygenes <- toupper(keygenes)

keygenes %in% rownames(GSE60993)

data <- as.data.frame(t(GSE60993)[,keygenes])

data <- log2(data+1)

data <- as.data.frame(cbind(type = GSE60993_meta[rownames(data), "group"],

data))

data$type <- factor(data$type, levels = c("IM", "Normal"))

roc_list <- c()

AUC <- c() # 0.670 0.615 0.440 0.648 0.764 0.808 0.868

for (i in keygenes) {

logistic_temp <- glm(as.formula(paste("type ~",i)), family=binomial(link='logit'), data=data)

temp_prob <- predict(logistic_temp,newdata=data, type="response")

temp_predict_type <- ifelse(temp_prob > 0.5, "IM", "Normal")

temp_predict_type <- factor(temp_predict_type, levels = c("IM", "Normal"), labels = c(1,0))

temp_predict_type <- as.numeric(temp_predict_type)

type_original <- factor(as.character(data$type), levels = c("IM", "Normal"), labels = c(1,0))

type_original <- as.numeric(as.character(type_original))

pred_roc <- prediction(temp_prob, type_original)

roc <- roc(type_original, temp_prob)

# === 新增 CI 计算代码 ===

auc <- round(auc(roc), 3) # 直接从roc对象中取值更稳妥

ci_res <- ci(roc) # 计算95% CI

ci_lower <- round(ci_res[1], 3)

ci_upper <- round(ci_res[3], 3)

label_text <- paste0("AUC = ", auc, "\n95% CI: ", ci_lower, "-", ci_upper)

# =======================

AUC <- c(AUC, auc)

gg <- ggroc(roc,color = "red", linetype = 1, size = 1, alpha = 1, legacy.axes = T) +

geom_abline(intercept = 0, slope = 1, color = "grey", size = 1, linetype = 1) +

labs(x = "False Postive Rate", y = "True Positive Rate") +

# === 修改这里的 annotate ===

annotate("text", x = 0.65, y = 0.25, # x和y微调了一下，给两行文字留空间

label = label_text, # 传入拼接好的AUC和CI文本

size = 5) + # 字体稍微调小一点防止出框

# ==========================

coord_cartesian(xlim = c(0,1), ylim = c(0,1)) +

theme_bw() +

theme(panel.background = element_rect(fill = "transparent"),

axis.ticks.length = unit(0.4,"lines"),

axis.ticks = element_line(color = "black"),

axis.line = element_line(size = 0.5, colour = "black"),

axis.title = element_text(colour = "black", size = 13, face = "plain"),

axis.text = element_text(colour = "black", size = 13, face = "plain")) +

ggtitle(i) + theme(plot.title = element_text(hjust = 0.5, size = 25))

roc_list <- c(roc_list, list(gg))

}

names(roc_list) <- keygenes

All_AUC <- data.frame(genes = keygenes,

auc = AUC)

All_AUC <- All_AUC[order(All_AUC$auc, decreasing = T),]

pdf("GSE60993_7_genes_ROC2.pdf", width = 20, height = 12)

plot_grid(roc_list[["GRN"]], roc_list[["IGF1"]], roc_list[["IL18"]], roc_list[["ITGB2"]],

roc_list[["NCF2"]], roc_list[["NCF4"]], roc_list[["SPP1"]],

nrow = 2, ncol = 4)

dev.off()

###

logistic_temp <- glm(as.formula(paste("type ~",paste(keygenes,collapse = " + "))), family=binomial(link='logit'), data=data)

temp_prob <- predict(logistic_temp,newdata=data, type="response")

temp_predict_type <- ifelse(temp_prob > 0.5, "IM", "Normal")

temp_predict_type <- factor(temp_predict_type, levels = c("IM", "Normal"), labels = c(1,0))

temp_predict_type <- as.numeric(temp_predict_type)

type_original <- factor(as.character(data$type), levels = c("IM", "Normal"), labels = c(1,0))

type_original <- as.numeric(as.character(type_original))

pred_roc <- prediction(temp_prob, type_original)

roc <- roc(type_original, temp_prob)

# === 新增 CI 计算代码 ===

auc <- round(auc(roc), 3)

ci_res <- ci(roc)

ci_lower <- round(ci_res[1], 3)

ci_upper <- round(ci_res[3], 3)

label_text <- paste0("AUC = ", auc, "\n95% CI: ", ci_lower, "-", ci_upper)

# =======================

AUC <- c(AUC, auc)

gg <- ggroc(roc,color = "red", linetype = 1, size = 1, alpha = 1, legacy.axes = T) +

geom_abline(intercept = 0, slope = 1, color = "grey", size = 1, linetype = 1) +

labs(x = "False Postive Rate", y = "True Positive Rate") +

# === 修改这里的 annotate ===

annotate("text", x = 0.65, y = 0.25,

label = label_text,

size = 5) +

# ==========================

coord_cartesian(xlim = c(0,1), ylim = c(0,1)) +

theme_bw() +

theme(panel.background = element_rect(fill = "transparent"),

axis.ticks.length = unit(0.4,"lines"),

axis.ticks = element_line(color = "black"),

axis.line = element_line(size = 0.5, colour = "black"),

axis.title = element_text(colour = "black", size = 13, face = "plain"),

axis.text = element_text(colour = "black", size = 13, face = "plain")) +

ggtitle("All Genes") + theme(plot.title = element_text(hjust = 0.5, size = 25))

pdf("GSE60993_all_genes_ROC2.pdf", height = 5, width = 5)

gg

dev.off()

##################################

# 加载必要包

library(pROC) # ROC分析

library(ggplot2) # 高级绘图

# 1. 准备数据

# 假设数据框名为data，第一列是type，其余列为基因表达量

# 创建二分类响应变量（0=Normal, 1=IM）

response <- as.integer(data$type == "IM") # 确保IM作为阳性事件

# 2. 构建逻辑回归模型（组合7个基因）

model <- glm(response ~ .,

data = data[, -1], # 排除第一列type

family = binomial(link = "logit"))

# 3. 获取预测概率

pred_prob <- predict(model, type = "response")

# 4. 计算ROC曲线和AUC

roc_obj <- roc(response, pred_prob,

levels = c(0, 1), # 阴性=0, 阳性=1

direction = "<") # 概率越大越可能是阳性

# 5. 绘制ROC曲线

ggroc(roc_obj, legacy.axes = TRUE) +

geom_abline(slope = 1, intercept = 0, linetype = "dashed", color = "red") +

labs(title = paste0("Combined Genes ROC (AUC=", round(auc(roc_obj), 3), ")"),

x = "False Positive Rate (1-Specificity)",

y = "True Positive Rate (Sensitivity)") +

theme_minimal()

#ROC曲线分析显示AUC=1时，这是一个极其重要且需要谨慎解读的结果，尤其在您的小样本数据集（n=14）中。这表示模型完美区分了所有样本（7个Normal vs 7个IM）

# 过拟合风险（更可能！）

# 样本量太小（n=14） 而特征较多（7基因）

# 模型记住了噪声而非真实模式

# 验证方法（必须执行！）：

# 留一交叉验证（LOOCV）

library(caret)

ctrl <- trainControl(method = "LOOCV")

cv_model <- train(

x = data[,-1],

y = as.factor(response),

method = "glm",

family = "binomial",

trControl = ctrl

)

# 查看交叉验证的AUC

cv_auc <- roc(response, predict(cv_model, type = "prob")[,2])$auc

# 当留一交叉验证（LOOCV）后仍得到 cv_auc = 1 时，这是一个 极其显著且需要深入验证 的结果，表明：
